# Health risk assessment and geochemical characterisation of trace elements in street dust: a case study from a quarry-influenced urban area of Istanbul

**DOI:** 10.1007/s10653-026-03264-y

**Published:** 2026-05-23

**Authors:** Kadir Ulutaş, Hedanur Yıldız, Rabia Emlek, Şilan Tekin, Elif Yavuz, Didar Üçüncüoğlu, Emre Yücer, Enes Özkök, Orhan Sevimoğlu, Abdulkadir Keskin, Seda Uyar

**Affiliations:** 1 Department of Health Management, Faculty of Health Sciences, Istanbul Medeniyet University, Istanbul, Türkiye; 2 Food Engineering Department, Faculty of Engineering, Çankırı Karatekin University, Çankırı, Türkiye; 3 TOBB Vocational School of Technical Sciences, University of Karabük, Karabük, Türkiye; 4 Department of Environmental Engineering, Faculty of Engineering, University of Karabük, Karabük, Türkiye; 5 Department of Environmental Engineering, Faculty of Engineering, Gebze Technical University, Kocaeli, Türkiye; 6 Department of Statistics, Faculty of Engineering and Natural Sciences, Istanbul Medeniyet University, Istanbul, Türkiye

**Keywords:** Heavy metals, Quarry-influenced area, Street dust, Air quality, Health risk assessment, Public health management

## Abstract

Dust from intensive mining operations like quarrying is a major concern in urban areas and should be assessed from environmental and public health perspectives. This study investigates the geochemical properties of trace elements in street dust and their associated health risks, based on samples collected from 40 georeferenced sites across three densely populated districts of Istanbul (Sultangazi, Eyüp, and Gaziosmanpaşa) located near and affected by quarries. The concentration distribution of 21 elements (Si > Al > Fe > Mg > Na > Ti > Mn > Zn > Ba > Cu > Cr > V > Pb > Ni > Sb > Co > As > Cd > Hg > Mo > Sn) was determined using Inductively Coupled Plasma Optical Emission Spectrometry (ICP-OES), indicating that elements associated with natural sources are more dominant. The concentrations of potentially toxic elements exhibited wide spatial variability, with Co ranging from 6.14 to 4467.49 mg kg^−1^, Cr from 36.78 to 2933.88 mg kg^−1^, Zn from 47.75 to 3828.63 mg kg^−1^, Pb from 6.89 to 1166.41 mg kg^−1^, and As from 0.25 to 1032.63 mg kg^−1^. Pollution levels were assessed, revealing extremely high contamination levels for Co based on the enrichment factor (EF = 107.99) and for Sn based on the geo-accumulation index (Igeo = 8.73). Human health risk assessment identifies ingestion as the dominant exposure pathway, with non-carcinogenic risk exceeding acceptable thresholds for children, particularly for Co, Cr, and As which their total hazard index was 2.65E+01. Carcinogenic risk via inhalation remains within acceptable limits. The findings emphasize the dominant role of particulate matter from quarries in contributing to urban street dust pollution. Moreover, the high levels of elements classified as potentially toxic elements (PTEs) in road dust, combined with quarrying activities, can lead to the accumulation of heavy metals in environmental components, disrupting ecosystem balance and posing significant environmental and human health risks to communities living in these areas.

**Supplementary Information:**

The online version contains supplementary material available at 10.1007/s10653-026-03264-y.

## Introduction

Urban environments located near extractive activities are exposed to increased particulate pollution due to continuous mechanical disturbance of geological materials. For example, stone quarrying represents a significant anthropogenic source of mineral dust through blasting, crushing, material handling, and transportation processes (Bingül, 2020; Petavratzi et al., 2005). Dust generated during quarry operations can be transported beyond extraction boundaries and deposited within adjacent residential and commercial areas, increasing interaction between quarry-derived emissions and urban environments (Ambastha & Haritash, 2022; Bui et al., 2020). The research studied indicated that dust originating from quarrying and mining activities frequently exhibits elevated concentrations of potentially toxic elements, largely due to the mineralogical characteristics of the quarried materials, which were arsenic, chromium, nickel, lead, cobalt, manganese, and zinc are commonly identified in dust and soil matrices collected from environments influenced by quarry operations (Abu Khatita, 2024; Etim & Adie, 2012). Mechanical abrasion and fragmentation during extraction enhance the mobilization of metal-bearing particles and promote their redistribution across surrounding urban surfaces (Kasongo et al., 2024; Khan, 2025).

The increase in physical structures such as roads and residential areas for society and individuals is one of the most prominent features of sustainable urbanization (Wu et al., 2024). Consequently, rapid urbanization has led to an increase in the use and demand for construction materials such as aggregates (sand, gravel, and crushed stone) extracted from various mechanized and conventional quarries and used in the construction sector (Hemmler et al., 2025; Magnusson et al., 2015). However, it has been noted that dust from quarries located near cities potentially poses social, ecological, environmental, and health risks (Abouian Jahromi et al., 2020; Saka & Hashim, 2024).

The transport of pollutants in urban environments depends on meteorological and topographical conditions, and therefore street dust, by accumulating particles from different sources, is used as a reliable indicator of urban air quality and environmental pollution (Ulutaş, 2022). It has been reported that, in Istanbul, the presence of densely populated residential areas surrounding quarries within the study area, combined with the active operation of open-pit quarrying, leads to significant dust generation and transport, resulting in notable environmental impacts (Kuzu & Ergin, 2005). Road surfaces act as effective sinks for particles derived from traffic activity, industrial emissions, and nearby extractive operations (Yan et al., 2020). Studies conducted in traffic-dominated urban areas demonstrate pronounced spatial heterogeneity in metal concentrations, reflecting localized emission sources and land-use characteristics (Alharbi et al., 2020; Xiao et al., 2020). Additional particulate inputs from quarry activities are expected to modify street dust composition in urban districts where extractive operations coexist with transportation corridors (Hadzi et al., 2019; Nduka et al., 2023). Human exposure to pollutant containing-street dust has been examined through ingestion, inhalation, and dermal contact pathways, with children identified as a more vulnerable population due to higher dust ingestion rates and lower body mass (Benhaddya et al., 2016; Han et al., 2023). Investigations conducted in quarry and mining regions also document adverse health effects associated with prolonged dust exposure, including respiratory symptoms among exposed populations (Isara et al., 2016; Abouian Jahromi et al., 2020; Onyedikachi et al., 2018).

ICP-OES is widely used in environmental geochemistry for multi-element analysis of urban dust and soils, enabling contamination assessment, comparison with previous studies, and application of pollution indices and human health risk evaluation frameworks (Alharbi et al., 2020; Othman et al., 2020; Raj et al., 2017; Ulutaş, 2023a). Heavy metals cause toxic effects on body systems, and the level of toxicity is directly related to the daily intake amount (Ohiagu et al., 2022). The heavy metal content of the dust is important for human health (Jomova et al., 2025). The free radicals in biological systems, which damage proteins and DNA, are formed by the action of carcinogenic Nickel (Ni) and Arsenic (As) metal ions (Fu & Xi, 2020; Schiavo et al., 2023a). Lead has effects that significantly alter the immune response both in humans and in animals (Mishra, 2009). Chromium (CrVI) has a more carcinogenic effect than Cr(III) and causes the most common health problems, including lung and nasal ulcers and cancers, skin allergies, bronchial asthma, and reproductive and developmental problems. Because Cr is naturally carcinogenic, it can be fatal in excessive amounts in the body (Shekhawat et al., 2015). Cancer stem cells can form even when trace amounts of the elements Aluminum (Al), As, Cr, Ni, and Selenium (Se) enter the human body (Mishra et al., 2010). Istanbul represents a megacity where rapid urban expansion has resulted in close spatial interaction between residential districts, transportation networks, and quarry-related activities. Existing studies conducted in Istanbul primarily attribute street dust contamination to traffic emissions and industrial sources (Huang et al., 2022; Sevimoğlu & Kuzu, 2025). Several districts within the metropolitan area are located near active or abandoned stone quarries, creating complex exposure settings that are not adequately characterized in current urban dust studies. Evidence from Türkiye indicates that quarry-related activities can influence metal distributions in environmental media and associated exposure pathways, particularly in environments where extractive operations coexist with residential land use (Horasan et al., 2021; Turhan et al., 2026). Limited attention has been given to densely populated urban districts where quarry activities, traffic emissions, and residential functions overlap, leading to uncertainty in source attribution and exposure characterization (Pham et al., 2024).

Studies conducted across different districts of the city document heavy metal contamination in street dust and urban soils, with contamination patterns largely attributed to traffic density and industrial activity (Yetimoğlu et al., 2007). In the study conducted on the E-5 Highway in Istanbul, heavy metal pollution was highlighted (Sezgin et al., 2004), and it was determined that traffic is the main source of heavy metal pollution despite the presence of industrial zones (Guney et al., 2010). It was found that metals originating from traffic (lead, copper, zinc) have a significant impact on health risks (Hou et al., 2019), and the necessity of controlling environmental pollution caused by increasing industrialization and traffic in Istanbul was emphasized (Yetimoğlu et al., 2007). However, the lack of quantitative assessments focusing on urban areas with high population density and intensive quarrying activities can be considered a major gap for Istanbul. In this context, Sultangazi and its neighboring districts, located in the northern part of Istanbul, exemplifies this urban configuration. The district integrates residential areas, transportation corridors, commercial zones, and quarry-related activities within a confined urban space. Rapid population growth has intensified environmental pressures and raised concerns regarding dust exposure and potential health risks among residents.

Quarries operating in close proximity within a confined area are causing significant disturbance to local residents, particularly in the district centers of Sultangazi and Gaziosmanpaşa, due to the dust and noise they generate. Weather conditions such as strong winds can further exacerbate these effects, at times reaching levels that pose a threat to public health. In this context, this study aims to investigate trace element contamination and assess the associated human health risks of potentially toxic elements (PTEs) in a quarry-influenced urban district of Istanbul. Street dust serves as a valuable indicator, as it accumulates particulate matter from multiple natural and anthropogenic sources over time, providing integrated insight into environmental pollution. Thus, the concentrations of heavy metals in street dust and to assess the associated human health risks of PTEs in a quarry-influenced urban three districts (Sultangazi, Eyüp, and Gaziosmanpaşa). In this study, 21 trace elements (Aluminum (Al), Arsenic (As), Barium (Ba), Cadmium (Cd), Cobalt (Co), Chromium (Cr), Copper (Cu), Iron (Fe), Mercury (Hg), Magnesium (Mg), Manganese (Mn), Molybdenum (Mo), Sodium (Na), Nickel (Ni), Lead (Pb), Antimony (Sb), Silicon (Si), Tin (Sn), Titanium (Ti), Vanadium (V), and Zinc (Zn)) were analyzed in samples taken from 40 different points using ICP-OES. The findings enable a quantitative assessment of the environmental impacts of quarrying activities and offer a comparative perspective with existing studies on heavy metal pollution observed in urban dust and quarry-affected environments. Metal contamination levels were evaluated through enrichment factors (EFs), and geo-accumulation index (Igeo), while potential anthropogenic and natural sources were identified using correlation and cluster analyses. Spatial distributions of contamination were visualized using Geographic Information System (GIS) techniques. Human health risks for different age groups were assessed following the United States Environmental Protection Agency (USEPA) methodology. The integrated approach adopted in this study provides a comprehensive assessment of metal pollution in a quarry-affected urban setting, contributes to improved understanding of environmental pressures associated with quarrying activities, and supports future urban environmental assessment and public health management efforts.

## Materials & methods

### Study area and dust sampling

**Fig. 1:**
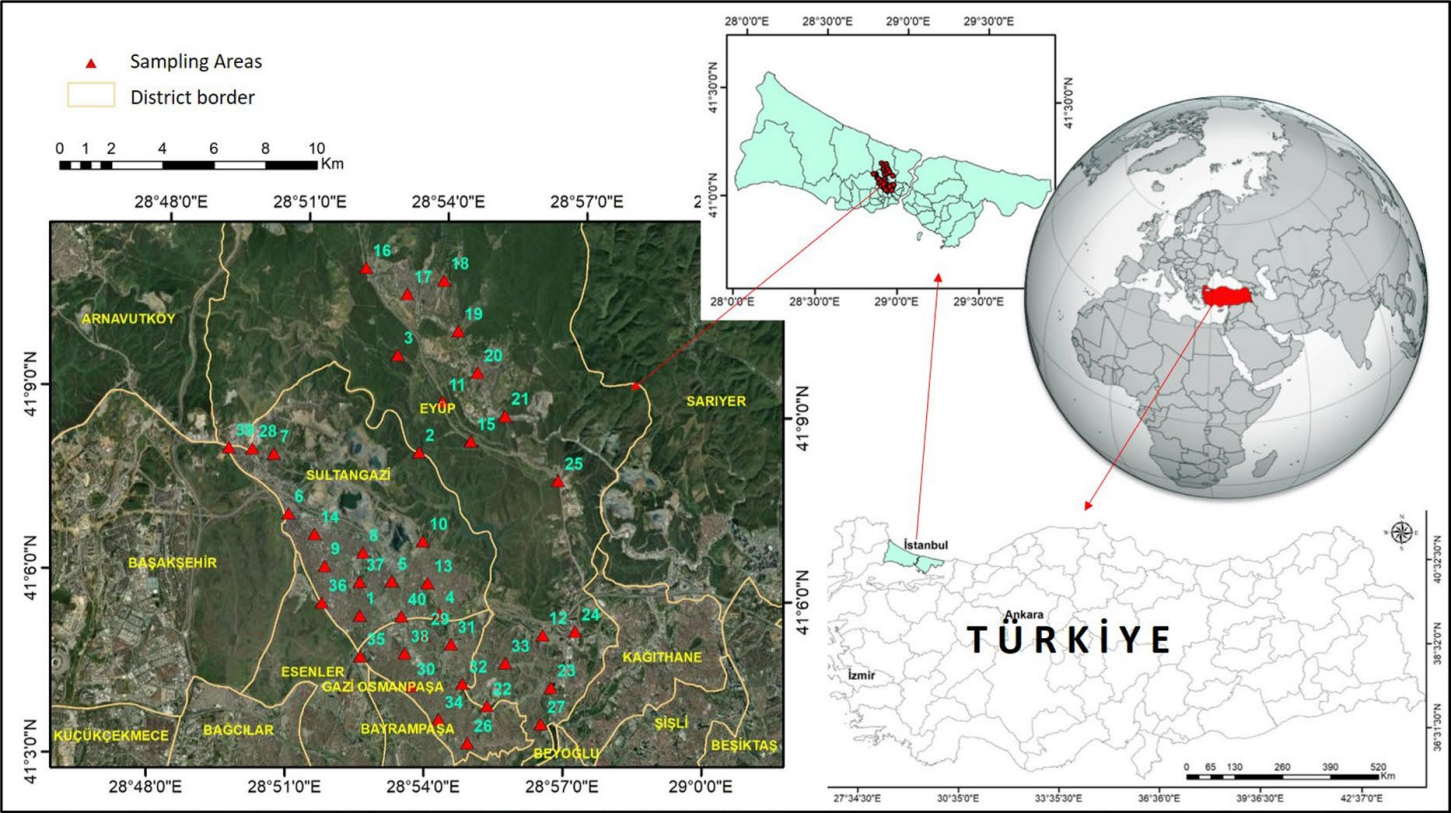
Spatial distribution of street dust sampling locations in the Sultangazi and its neighboring districts, Istanbul, Türkiye

This study was conducted in the Sultangazi and its neighbouring districts in the northern part of European side of Istanbul, Türkiye (Fig. 1). The study includes key features such as the Alibey Dam Lake, the Mimar Sinan Urban Forest, and the Cebeci quarries. The Cebeci region (41.1209° N, 28.8766° E) is characterized by its well known Carboniferous limestone deposits, which are extracted for aggregate production (Er et al., 2017). Cebeci Quarries supply a substantial share of the stone demand on the European side of Istanbul, with approximately 30 million tons of limestone and sandstone extracted annually. While limestone is utilized to its finest fraction, greywacke–shale is not fully exploited. The sandstone is processed into various sizes (grain diameter > 5 mm) and used as asphalt and concrete aggregate. Intensive quarry operations to support numerous construction projects result in heavy traffic and considerable dust emissions from transportation, with dispersion governed by wind speed and direction. The O-7 Northern Marmara Highway, a transit corridor linking the European and Asian continents, runs adjacent to the quarry. During sampling on June 2–3, 2024, the prevailing wind direction was identified as southwest based on HYSPLIT back-trajectory analysis (Fig. S1a-b).

The study area is characterized by high population density and intensive urban activities. Residential neighborhoods are closely associated with commercial zones and major transportation corridors. Areas influenced by extractive industrial activities are also present. Active and abandoned stone quarry sites occur near residential areas and road networks. This spatial configuration favors the accumulation of quarry-derived particulate matter on urban surfaces.

Street dust samples were collected from 40 locations across the study area to represent different land use characteristics and traffic conditions on June 2–3, 2024. The samples were collected as follows: 15 from Sultangazi, 8 from Gaziosman paşa, and 17 from Eyüp districts. Sampling points were selected from sidewalks and road edges that were free from recent disturbances. Loose surface dust was collected using a clean plastic brush and polyethylene tray. The collected material was air-dried at room temperature. The sieving procedure was performed using a sieve, corresponding to a particle size fraction smaller than 150 µm. The sieved material was homogenized and stored in polyethylene containers prior to chemical analysis.

### Analytical procedures

All samples underwent overnight drying at 120 °C for 24 h before digestion for elemental analysis. For microwave-assisted acid digestion, 0.25 g of each dried and sieved sample was weighed and placed into a PTFE test tube, followed by the addition of 8 mL nitric acid (HNO₃, 65%; Merck Suprapure), 2 mL hydrofluoric acid (HF, 48%; Merck Suprapure), and 1 mL perchloric acid (HClO₄; Merck Suprapure) (Suryawanshi et al., 2016; Ulutaş, 2022). Samples were digested at 140 °C for 60 min in a 1200 W microwave system (Milestone Inc.), then cooled for 45 min and diluted to 50 mL with ultrapure water (18.3 MΩ). After cooling, the samples were filtered through Whatman No. 12 filter paper (0.45 µm, 11 mm) and subsequently diluted to 50 mL with distilled water in a volumetric flask. The solutions were stored in the refrigerator until analysis.

Elemental concentrations were measured by inductively coupled plasma–optical emission spectrometry (ICP–OES) using a PerkinElmer Avio 200 instrument. Standard stock solutions (1000 mg/L Sigma Aldrich and Merck) were used to prepare calibration standards. All analyses were conducted in triplicate (n = 3), and mean values were reported. Certified reference material (BCR-723 IRMM JRC Road Dust, European Commission) was analyzed to evaluate method accuracy and precision. Recovery rates, certified values, and detection limits are summarized in Table S1**.**

### Spatial analysis

GIS techniques were applied to evaluate the spatial variability of heavy metal concentrations across the study area. Sampling locations were georeferenced using recorded geographic coordinates. GIS-based spatial distribution maps were generated to visualize concentration patterns of individual metals. Spatial interpolation was conducted to estimate metal concentrations between sampling points. This approach supported the identification of localized contamination hotspots and spatial trends within the study area. The generated maps were used to interpret contamination patterns in relation to land-use characteristics, traffic density, and quarry-influenced zones. Spatial analysis has been widely applied in urban dust studies to assess spatial heterogeneity and potential source-related patterns (Huang et al., 2022; Lima et al., 2023).

### Statistical analysis

Statistical analyses were performed to investigate distribution characteristics, inter-element relationships, and spatial heterogeneity of heavy metals in street dust samples. Prior to advanced statistical modeling, data preprocessing steps were undertaken. This included screening the dataset for missing values and extreme outliers. To minimize scale-related bias and ensure equal contribution of all elements in multivariate models, raw element concentrations were standardized using z-score transformation. Descriptive statistical parameters, including mean, median, standard deviation, minimum, maximum, skewness, and kurtosis, were calculated to characterize the dataset by using ANOVA software (Sharma & Ojha, 2019).

The underlying assumptions for statistical tests were strictly evaluated prior to analysis. Since the Shapiro–Wilk test showed that the assumption of normality was not met (*p* < 0.05), non-parametric alternatives were selected as they do not require normally distributed data. Spearman rank correlation analysis was conducted to assess inter-element relationships and to identify metals with potential common sources or similar geochemical behavior. Correlation analysis is widely used in the literature to identify inter-element relationships and potential common sources (Acosta et al., 2011; Ulutaş et al., 2026).

For multivariate pattern recognition, specific techniques were chosen based on their robust performance with complex, non-normal environmental data. Hierarchical cluster analysis (HCA) was applied to classify sampling locations based on similarities in metal concentration patterns, as it effectively reveals hidden, nested structures within data without requiring a predefined number of clusters (Tokalıoğlu & Kartal, 2006). Two-way HCA was used to classify the sampling points according to their pollution profiles, based on the Ward linkage method and squared Euclidean distance (Ulutaş et al., 2025). Clustering results were visualized using a dendrogram and a heatmap. Furthermore, Principal Component Analysis (PCA) was applied to identify the latent sources of heavy metals and reduce the dimensionality of the dataset, providing a strong mathematical validation of the identified pollution profiles. Differences in metal concentrations among identified clusters were evaluated using the non-parametric Kruskal–Wallis H test (Borge et al., 2022). Effect size values (ε^2^) were calculated to quantify the magnitude of observed differences among clusters. Statistical significance was evaluated at *p* < 0.05. Descriptive statistical parameters and cluster analysis were performed using Orange software version 3.4. Other statistical and visual analyses were performed using R Studio.

### Pollution indices

Metal contamination levels were evaluated using commonly applied pollution indices, including the enrichment factor (EF) and geo-accumulation index (I*geo*). EF was calculated to assess the degree of anthropogenic influence on heavy metal concentrations using Eq. (1), as commonly applied in contamination assessment studies (Glasby & Szefer, 1998; Khundrakpam et al., 2025; Taghavi et al., 2019).

1 $${\mathrm{EF}} = \frac{{\left( {\frac{{C_{i} }}{{C_{ref} }}} \right)_{sample} }}{{\left( {\frac{{C_{i} }}{{C_{ref} }}} \right)_{crust} }}$$

where *C*_*i*_ represents the concentration of metal *i* in the street dust sample and *C*_*Al*_ represents the concentration of Al used as the reference element (Farahi et al., 2025; Taghavi et al., 2019). Al was selected as the reference element based on its crustal origin, geochemical stability, and frequent application in urban dust studies. EF values were interpreted as EF < 2 (minimal enrichment), 2 ≤ EF < 5 (moderate enrichment), 5 ≤ EF < 20 (significant enrichment), 20 ≤ EF < 40 (very high enrichment), and EF ≥ 40 (extremely high enrichment).

The geo-accumulation index (I*geo*) was calculated following the method proposed by Müller (1969) using Eq. (2):

2 $${\mathrm{I}}_{{{\mathrm{geo}}}} = {\mathrm{log}}_{2} \frac{{{\mathrm{C}}_{i} }}{{1.5{ } \times {\text{ C}}_{i, background} }}$$

where *Ci* is the measured concentration of metal* i* in street dust and C*i*, *background* is the corresponding background concentration. The constant factor 1.5 was introduced to account for possible natural variations in background values caused by lithogenic effects. I*geo* values were classified into seven contamination classes ranging from uncontaminated (I*geo* ≤ 0) to extremely contaminated (I*geo* ≥ 5).

### Human health risk assessment

Human health risk assessment was conducted following methodologies developed by the United States Environmental Protection Agency (USEPA, 1989; USEPA, 2002). Potential non-carcinogenic and carcinogenic health risks associated with exposure to PTEs in street dust were evaluated separately for adults and children. In this study, health risk assessment was conducted based on the PTEs contents of Co, Cr, Cu, Mn, Ni, Pb, and Zn in street dust. The average daily intake (ADI) values for ingestion, inhalation, and dermal contact routes were calculated using Eqs. 3–5. Table S2 shows explanations of the parameters needed in the calculations. Additionally, Table S3 lists the reference dose (RfD) and cancer slope factor (CSF) values required in assessments of hazard index (HI) and cancer risk (CR).

3 $${\mathrm{ADI}}_{{{\mathrm{ing}}}} = {\text{C }} \times \frac{{{\text{ingR }} \times {\text{ EF }} \times {\text{ ED}}}}{{{\text{BW }} \times {\text{ AT}}}}{ } \times { }10^{ - 6} { }$$

4 $${\mathrm{ADI}}_{{{\mathrm{inh}}}} = {\text{C }} \times \frac{{{\text{inhR }} \times {\text{ EF }} \times {\text{ ED}}}}{{{\text{PEF }} \times {\text{ BW }} \times {\text{ AT}}}}$$

5 $${\mathrm{ADI}}_{{{\mathrm{der}}}} = {\text{C }} \times \frac{{{\text{SL }} \times {\text{ SA }} \times {\text{ ABS }} \times {\text{ EF }} \times {\text{ ED}}}}{{{\text{BW }} \times {\text{ AT}}}}{ } \times { }10^{ - 6}$$

Non-carcinogenic health risk was evaluated for ingestion, inhalation, and dermal contact routes using the hazard quotient (HQ) and hazard index (HI), as follows (Eqs. 6–7):

6 $${\mathrm{HQ}}_{{{\mathrm{ing}},{\text{ inh}},{\text{ der}}}} = \frac{{{\mathrm{ADI}}_{{{\mathrm{ing}},{\mathrm{inh}},{\mathrm{der}}}} }}{{{\mathrm{RfD}}}}$$

7 $${\mathrm{HI}} = \sum {\mathrm{HQ}}_{{{\mathrm{ing}},{\text{ inh}},{\text{ der}}}}$$

HI values reflect the combined non-carcinogenic risk from ingestion, inhalation, and dermal contact pathways for each element. An HI value greater than 1 indicates potential non-carcinogenic health risk.

CR was estimated using Eqs. 8 and 9 for only inhalation route.

8 $${\mathrm{LADD}} = \frac{{{\text{C }} \times {\text{ EF}}}}{{{\text{AT }} \times {\text{ PEF}}}}{ } \times \left( {\frac{{{\mathrm{Inh}}_{{{\mathrm{child}}}} { } \times {\text{ ED}}_{{{\mathrm{child}}}} }}{{{\mathrm{BW}}_{{{\mathrm{child}}}} }} + \frac{{{\mathrm{Inh}}_{{{\mathrm{adult}}}} { } \times {\text{ ED}}_{{{\mathrm{adult}}}} }}{{{\mathrm{BW}}_{{{\mathrm{adult}}}} }}} \right)$$

9 $${\mathrm{CR}} = {\text{LADD }} \times {\mathrm{SF}}$$

Carcinogenic risk values below 1.0E−06 were considered acceptable, between 1.0E−06 and 1.0E−4 were considered tolerable, while values exceeding 1.0E−04 indicate an elevated cancer risk, according to USEPA guidelines (USEPA, 1998).

## Results and discussion

### Heavy metal concentrations and descriptive statistical characteristics of street dust

Heavy metal concentrations determined in street dust samples collected from study area are presented in Table 1. 21 elements were quantified across 40 sampling locations. Si, Al, and Fe showed the highest mean concentrations, with average values of 83,541.45 mg kg^−1^, 21,843.18 mg kg^−1^, and 17,346.24 mg kg^−1^, respectively. Na, Mg, and Mn were also detected at elevated concentration levels, with mean concentrations of 13,792.36 mg kg^−1^, 5,630.45 mg kg^−1^, and 3,425.68 mg kg^−1^, respectively. These elements are typically associated with crustal material and urban background inputs, and their elevated levels reflect the combined influence of natural lithogenic sources and diffuse urban activities (Balabanova et al., 2017). Potentially toxic elements showed a wide range of mean concentrations. Co exhibited a notably high mean concentration of 432.64 mg kg^−1^. Mean concentrations of Zn, Cr, Cu, Pb, and As were 384.08 mg kg^−1^, 298.17 mg kg^−1^, 231.68 mg kg^−1^, 128.38 mg kg^−1^, and 66.27 mg kg^−1^, respectively. Cd, Hg, and Mo were recorded at lower concentration levels, with mean values below 1 mg kg^−1^. The concentration levels of Co, Zn, Cr, Pb, and As summarized in Table 1 indicate pronounced anthropogenic enrichment, which has been widely attributed to traffic-related abrasion processes and localized industrial inputs in densely populated urban environments (Taiwo et al., 2020). Comparable enrichment patterns for Zn, Cr, Pb, and As have been reported in urban street dust from densely populated city centers characterized by mixed emission sources (Díaz-Rizo et al., 2023).

**Table 1:** Descriptive statistics of heavy metals in street dust (mg kg^−1^)

Element	N	Mean	Median	SD	Min	Max	Skewness	Kurtosis	*p*
Si	40	83,541.45	59,460.31	79,792.77	615.14	343,136.93	1.32	2.11	< 0.001
Al	40	21,843.18	16,685.63	16,582.87	3566.33	79,708.59	2.08	4.72	< 0.001
Fe	40	17,346.24	16,986.84	12,227.77	123.62	57,654.33	1.21	2.89	< 0.001
Mg	40	5630.45	4622.58	4390.57	438.60	17,162.06	0.86	0.12	0.004
Na	40	13,792.36	12,562.92	6336.38	3540.62	35,276.69	1.88	4.88	< 0.001
Ti	40	1841.02	1618.96	938.32	713.91	5347.48	2.31	6.16	< 0.001
Mn	40	3425.68	3864.15	3155.88	41.50	12,863.40	0.70	0.15	< 0.001
Zn	40	384.08	171.69	678.20	47.75	3828.63	4.00	17.85	< 0.001
Ba	40	270.33	289.69	97.76	80.30	599.18	0.47	2.21	0.032
Cu	40	231.68	74.38	408.63	24.81	1953.04	2.91	8.78	< 0.001
Cr	40	298.17	63.53	656.52	36.78	2933.88	2.83	7.41	< 0.001
V	39	49.42	49.42	17.10	5.67	102.80	0.45	2.26	0.154
Pb	40	128.38	20.95	286.37	6.89	1166.41	2.66	6.10	< 0.001
Ni	40	37.45	29.00	23.93	2.74	144.42	2.72	9.95	< 0.001
Sb	35	2.40	2.04	1.44	0.11	5.91	0.84	0.60	0.019
Co	40	432.64	10.32	1175.47	6.14	4467.49	2.69	6.14	< 0.001
As	40	66.27	6.26	193.87	0.25	1032.63	3.92	16.74	< 0.001
Cd	40	0.46	0.10	1.21	0.01	5.86	3.66	13.23	< 0.001
Hg	40	0.06	0.02	0.13	0.00	0.64	3.52	12.64	< 0.001
Mo	35	0.17	0.17	0.06	0.06	0.35	0.88	1.97	0.074
Sn	38	47.21	11.61	106.65	0.07	462.04	2.94	8.14	< 0.001

Concentrations are expressed in mg kg^−1^. N represents the number of samples. N values lower than 40 reflect elements with non-detects at some locations. SD denotes standard deviation. Shapiro–Wilk* p* values indicate normality test results

*p* < 0.05 indicates deviation from normal distribution

The exceptionally high Co concentrations observed in the study area require additional consideration. Co commonly occurs in mafic and mixed lithological formations and can be released into the surrounding environment during quarrying activities through mechanical extraction, crushing, and material handling processes (Kasongo et al., 2024; Upadhyay, 2025). The wide concentration range recorded for Co, from 6.14 to 4,467.49 mg kg^−1^, together with its strong spatial variability, suggests localized inputs rather than uniform background enrichment. Strong positive correlations between Co and other quarry-associated elements, including Cr, Ni, As, and Pb, further support a common source linked to quarry-derived particulate matter. Elevated Co concentrations of similar magnitude have been reported in quarry- and mining-influenced environments, where repeated deposition and resuspension of mineral dust enhance surface accumulation (Bolaji et al., 2025).

Differences between mean and median values were observed for Zn, Pb, Cu, Cr, Co, and As, indicating asymmetric data distributions. Such asymmetry reflects localized enrichment rather than uniform background accumulation, as commonly reported for street dust in traffic-intensive urban districts (Skorbiłowicz et al., 2023). Ba, V, and Mo showed relatively close mean and median values. Variability levels differed among elements. High standard deviation values were recorded for Si, Al, Fe, Zn, Co, Cr, and Cu, whereas lower variability was observed for Cd, Hg, and Mo. Wide concentration ranges reported in Table 1, including values of 47.75–3,828.63 mg kg^−1^ for Zn, 6.89–1,166.41 mg kg^−1^ for Pb, 36.78–2,933.88 mg kg^−1^ for Cr, and 6.14–4,467.49 mg kg^−1^ for Co, support strong spatial heterogeneity and point-scale contamination processes linked to episodic emissions and resuspension (Lippmann et al., 2015). The dataset exhibits significant heterogeneity, characterized by wide concentration ranges and high coefficients of variation. In particular, the significantly higher mean values compared to median values, together with positive skewness coefficients for anthropogenically derived toxic elements such as Zn, Pb, Cu, Cr, As, Cd, and Hg (Gope et al., 2017; Wei & Yang, 2010), indicate that pollution is not homogeneously distributed across the study area but is instead concentrated at specific points. For example, the difference between the mean (384.08 mg kg^−1^) and median (171.69 mg kg^−1^) for Zn, along with high kurtosis values, points to the presence of outliers and point sources of pollution. Distribution characteristics differed among the analyzed elements. Shapiro–Wilk test results are reported in Table 1 and indicate non-normal distributions for Si, Al, Fe, Na, Mg, Mn, Zn, Cu, Cr, Pb, Co, As, Cd, Hg, and Sn, with p values below 0.05. Normal distribution patterns were observed for V and Mo, with Shapiro–Wilk *p* values of 0.154 and 0.074, respectively.

The spatial distribution of elements Si, Al, Fe, Na, Mg, and Mn shows that these elements are predominantly associated with crustal materials and urban background inputs, and do not exhibit a significant divergence between different land-use types. This suggests that the concentrations of these elements largely reflect the combined effect of natural lithogenic sources and widespread urban activities. In contrast, the observed enrichment trends for elements Zn, Cr, Pb, and As are more pronounced, particularly in areas of intensive urban use, indicating the influence of mixed emission sources (traffic, industrial activities, etc.). The distribution of Co, unlike the other elements, shows the influence of local inputs related to proximity to the quarry rather than background levels. Overall, the relationship between element concentrations and land-use types and proximity to the quarry reveals spatially divergent effects of natural and anthropogenic resources.

In this study, V, Mo, Ba, Mn, Se, and Al were identified as the most abundant elements in the samples, particularly near the Hasdal-Kemerburgaz (D-20) road, which is classified as an urban road, and in proximity to a concrete plant. These findings indicate that traffic emissions and concrete production activities play a significant role in elevating the concentrations of these elements. In contrast, As, Ni, Sn, and Cu were dominant in street dust samples from residential and traffic-influenced areas, highlighting the effect of mixed urban land use. Notably, Cr, Co, Ti, S, Cd, Hg, and Pb were found at the highest levels in dust collected from a water aqueduct located within forested and urban forest areas. Although these locations are distant from direct anthropogenic sources, the presence of typically industrial elements suggests that long-range atmospheric transport and subsequent deposition may contribute to their accumulation, in addition to natural geological inputs. Furthermore, Zn showed the highest concentration in a schoolyard, while Sb was most elevated in a university campus parking lot, supporting its association with vehicular sources. Na and Mg were predominantly found in areas characterized by road traffic and residential buildings. Considering the sources where these elements, which were found at the highest levels compared to other samples, are present, these results demonstrate clear differences in elemental composition across land-use categories and indicate that road dust is influenced by both natural sources and anthropogenic activities, including traffic, industrial operations, and possible long-distance transport processes.

A comparative overview of selected heavy metal concentrations reported in samples from environments influenced by quarrying and related extractive activities is presented in Table 2. The higher values obtained in this study compared to six sample sets reported in the literature are thought to be attributable to factors such as the sampling technique employed, the size and number of samples, the larger sampling area, and the sensitivity of the measurement technique. In particular, coverage of a broad sampling area enhanced the representativeness of the study and contributed to the higher values obtained. Additionally, the larger sample size relative to previous studies and the use of a more sensitive and repeatable analytical method are considered important factors explaining the elevated values observed. The findings of the present study are therefore placed within a broader international context, while acknowledging differences in sampling design, dust particle size fractions, analytical approaches, and local geological conditions among studies; accordingly, direct quantitative equivalence between studies is not implied.

**Table 2:** Literature comparison of selected heavy metal concentrations (mg kg^−1^) in samples from quarry influenced environments

Elements	Turhan et al., (2026)	Damola et al., (2021)	Etim and Adie (2012)	Mellese et al., (2025)	Ajibade et al., (2022)	Adetola et al., (2024)	In this study
	XRF	ICP-OES	AAS	ICP-OES	ICP-MS	ICP-OES	ICP-OES
	126 samples	24 samples	6 samples	5 samples	24 samples	10 samples	40 samples
	Perlite samples	Street dust in a quarry	Topsoil samples within the vicinity of limestone quarry	Exploitation sites of sandstones quarry sites	Quarry soil-dust samples	Soils from quarry sıte	Street dust samples within the vicinity of a quarry
Ti	528.3	4010					
V	7.1				59.75	24.72	49.42
Cr	64.9	126	11.81	115.41	52.46	0.84	298.17
Mn	383.5	716	512.50			192	3425.68
Fe	6585.4	43,900	11,220.17			4354	17,346.24
Co	7.0	24	13.39		12.40	3.81	432.64
Ni	19.8	56	10.91	10.58	24.25	1.6	37.45
Cu	4.3	25	17.93	13.23	42.22	12.37	231.68
Zn	31.1	65	42.90	15.10	88.66	11.48	384.08
As	4.2	1.7			4.64	1.59	66.27
Pb	26.4	14.8	16.00	19.91	32.83	3.05	128.38
Zr	93.7						
Cd		0.1	1.15		0.11		0.46
Mo					1.31	0.69	0.17
U					6.97		
Th					34.42		
Sr					416.75		
Se					0.73	11.63	
Al		80,400				1678	21,843.18
Hg						1.51	0.06
Sb						5.52	2.4
Sn						3.6	47.21

Values reported for quarry influenced urban environments in selected international studies. Dust size fractions differ among studies and are reported where available. Concentrations are expressed as mg kg^−1^. Elements were selected according to their relevance to human health risk assessment discussed in the main text. NR indicates that the element was not reported

### Spatial distribution of heavy metals

**Fig. 2:**
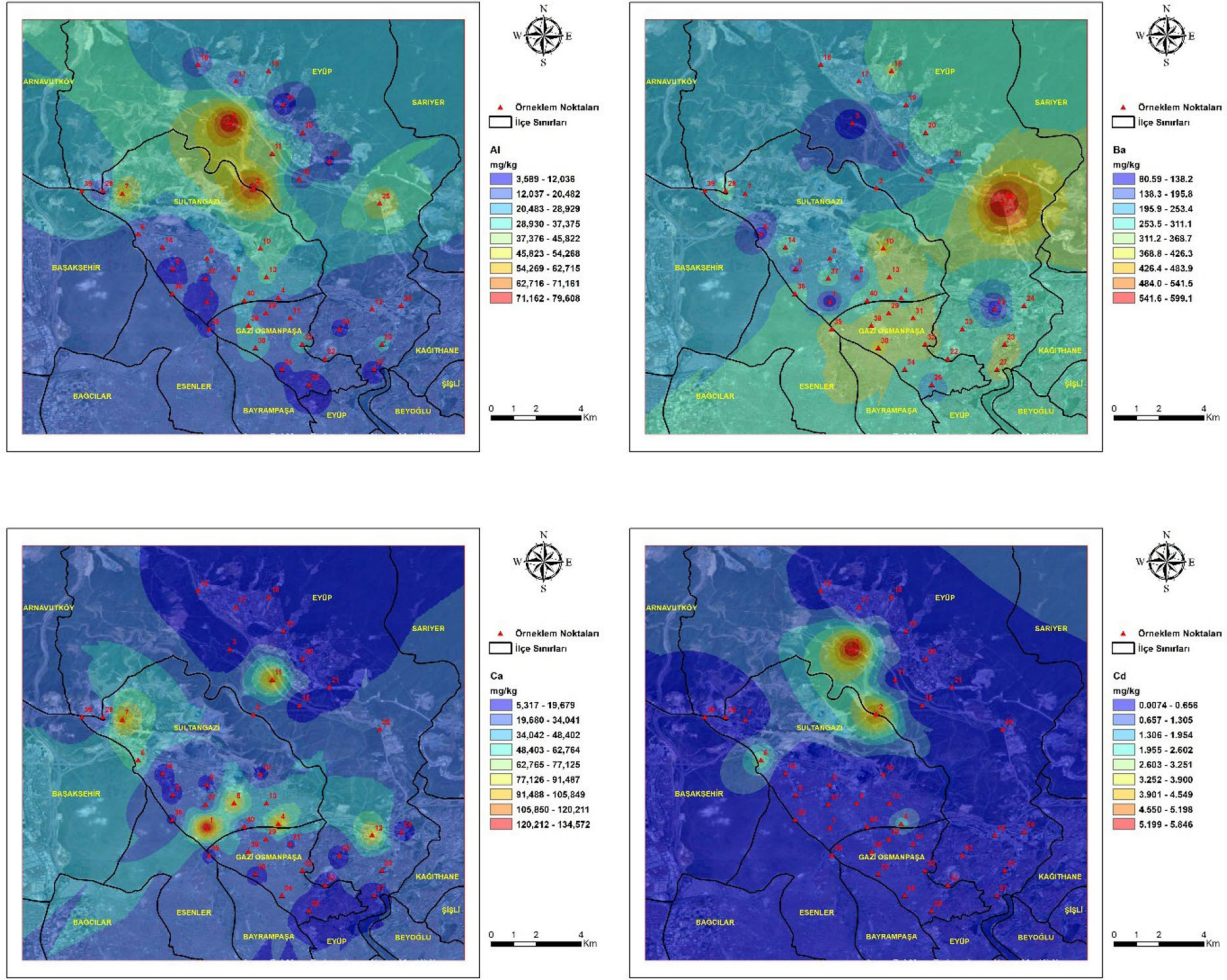

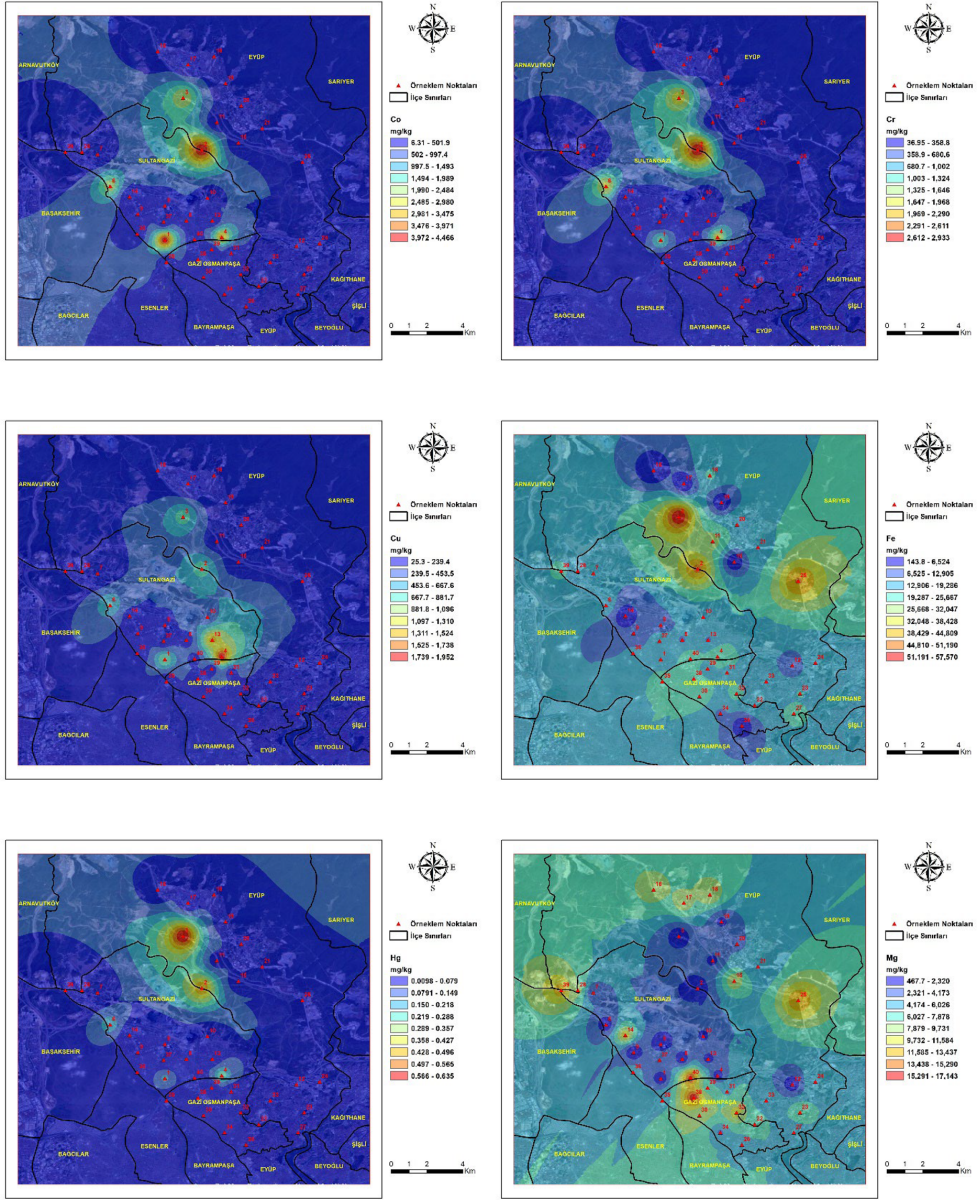

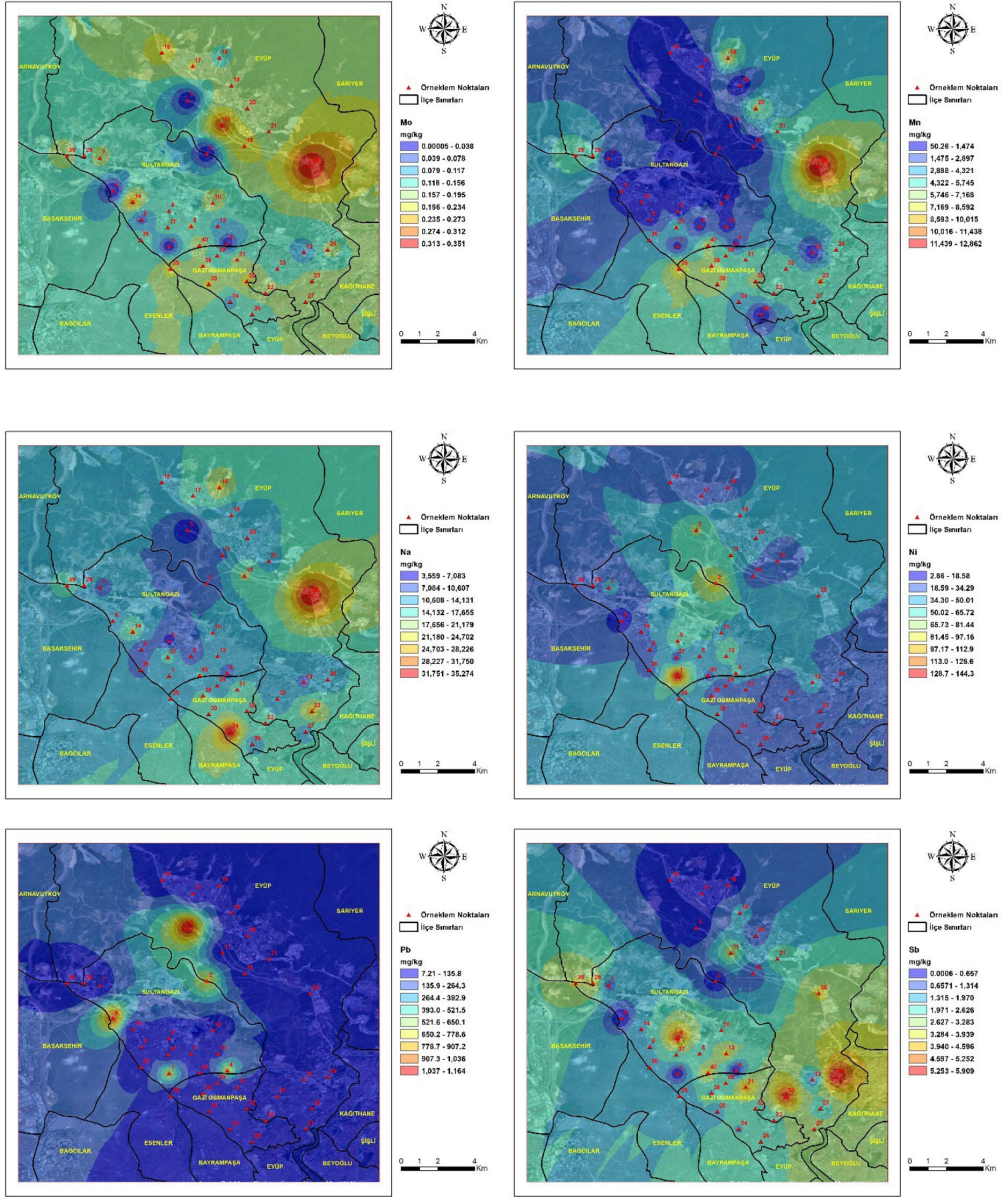

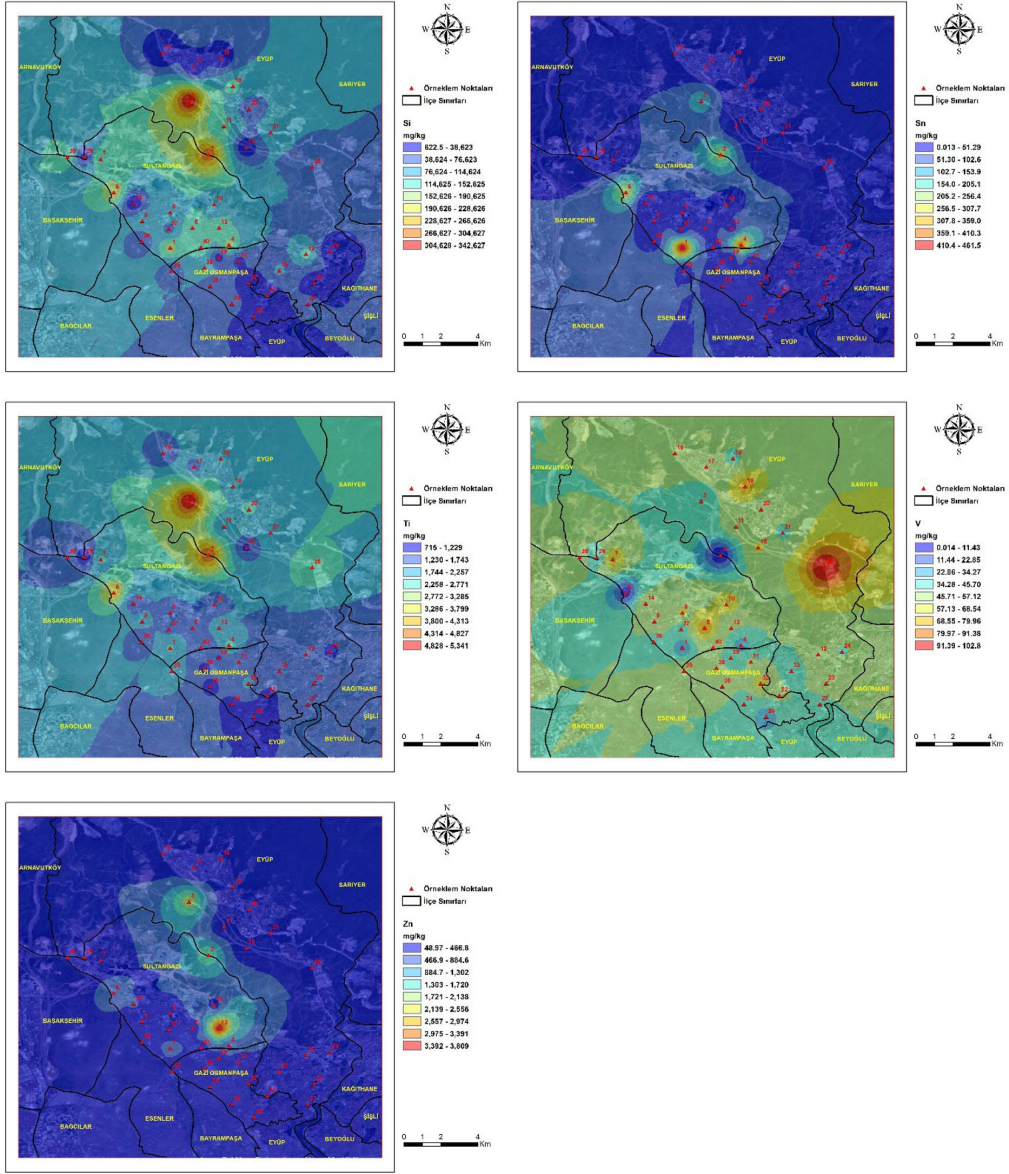
Spatial distribution maps of heavy metal concentrations in street dust samples collected from the study area. Maps illustrate the spatial distribution of Al, Cr, Cd, Cu, Hg, Co, Mn, Ni, Pb, Sn, Zn, V, Ba, As, Fe, Mg, and Na across the study area. Maps were generated using Geographic Information System techniques based on measured metal concentrations at sampling locations. Color gradients indicate relative concentration levels of each element across the study area

The spatial distribution of heavy metal concentrations in street dust samples from the study area is presented in Fig. 2**.** Spatial variation was observed across the study area for all analyzed elements. Pronounced spatial heterogeneity was identified for Zn, Pb, Cu, Cr, and Co, with elevated concentrations confined to specific sampling locations rather than being uniformly distributed across the district. This spatial heterogeneity indicates the dominance of localized emission sources and suggests that contamination is controlled by site-specific anthropogenic activities rather than diffuse urban background processes (Gope et al., 2017; Taiwo et al., 2020). As, Cd, and Hg showed localized areas with higher concentrations, indicating point-scale enrichment at a limited number of sites. Such enrichment patterns are characteristic of elements introduced through episodic emissions and subsequent surface accumulation in urban environments influenced by mixed traffic and industrial sources (Khan et al., 2023). Al, Fe, Na, Mg, and Mn showed wider spatial distributions across the study area, with gradual concentration gradients reflecting broader lithogenic and urban background contributions. The comparatively uniform spatial distribution of these elements supports their association with natural soil material and resuspended crustal components rather than localized anthropogenic inputs (Reimann & Caritat, 2005).

Spatial interpolation results reflected variation in metal concentrations within the study area. Distinct concentration hotspots were observed in zones characterized by intense traffic activity and proximity to quarry-influenced areas, whereas lower concentrations were generally recorded in predominantly residential zones with reduced anthropogenic disturbance. The spatial coincidence of elevated metal concentrations with traffic corridors and quarry-influenced zones, as illustrated in Fig. 2, indicates that vehicular activity and quarry-derived particulate emissions jointly influence the observed spatial patterns (Peng et al., 2016). Similar hotspot configurations linked to the combined influence of traffic flow and quarry proximity have been reported in urban environments adjacent to extractive activities (Rusakova et al., 2025). Comparable hotspot configurations have been reported in urban districts where traffic intensity overlaps with extractive industrial activities, leading to enhanced accumulation of metal-bearing particles on surrounding surfaces (Song et al., 2025). The contrast between hotspot areas and residential zones further emphasizes the role of land-use characteristics in shaping the spatial variability of street dust contamination (Ren et al., 2024).

### Spatial clustering

Multidimensional Scaling (MDS) analysis was applied to visualize the similarities in the chemical compositions of dust samples collected from the study area and to group sampling points according to their elemental content (Gunawardana et al., 2012). To prevent errors that might arise from unit differences in elemental concentrations in the dataset, the data was standardized by applying a Z-score transformation to all variables before the analysis. Euclidean distance was used as the distance measure. The goodness-of-fit and reliability values of the model obtained from the analysis are presented in Table 3.

**Table 3:** MDS Model Summary and Fit Values

Dimension	Cronbach’s Alpha	Eigenvalue	Explained variance
1	0.970	13.062	%79
2	0.745	3.442	%21
Total	0.986	16.503	%100

**Fig. 3:**
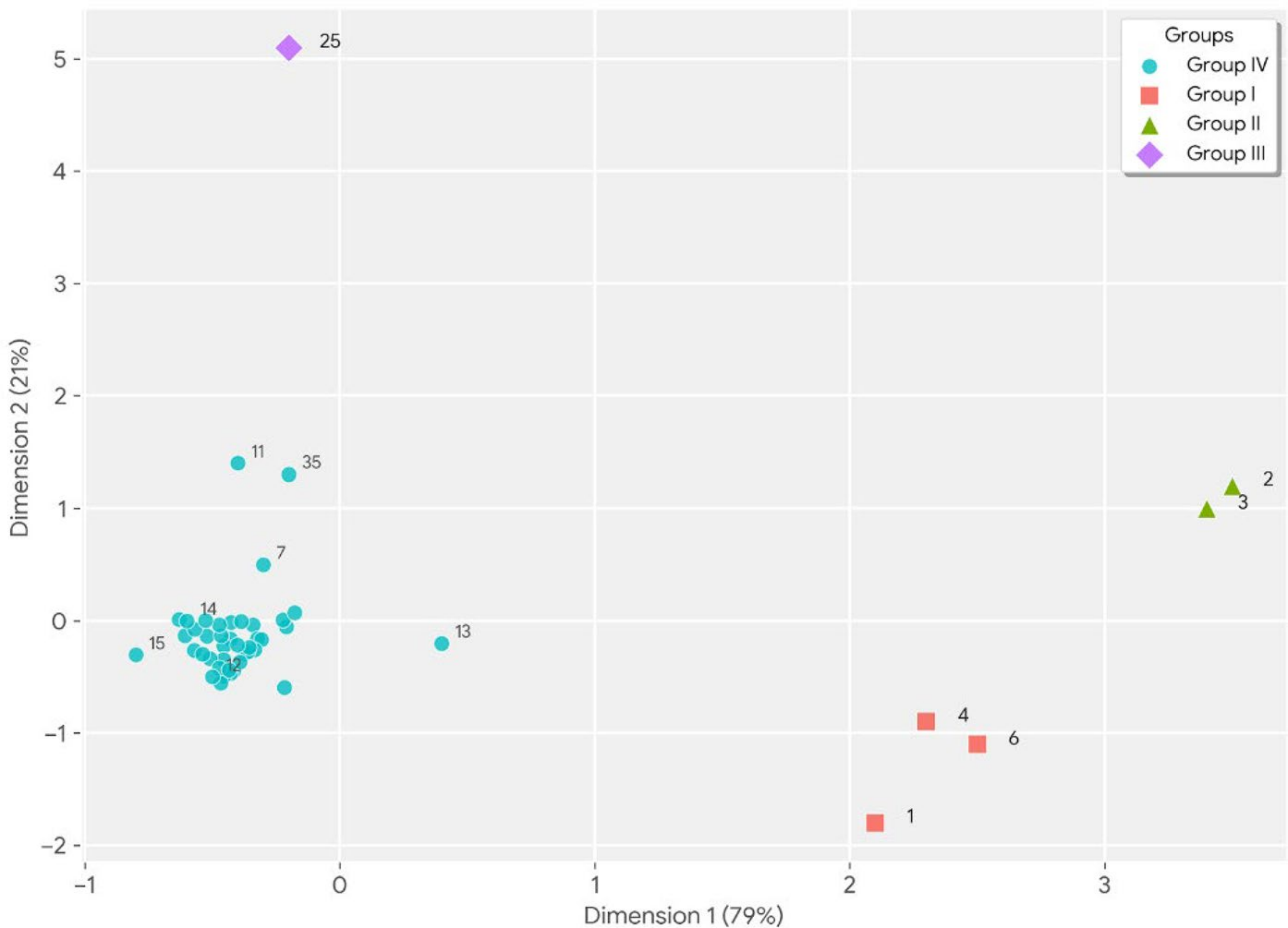
Two-Dimensional Configuration Map of the Elements (Euclidean Distance Model)

According to the analysis findings, the total Cronbach’s Alpha coefficient of the two-dimensional solution was calculated as 0.986. Considering the literature where statistically significant values above 0.70 are considered reliable, this value indicates that the model’s internal consistency and data representation power are at an excellent level. The model’s total eigenvalue (16.503) proves that a very large portion of the variation between samples is successfully explained by this two-dimensional map. The spatial distributions of the samples in the obtained two-dimensional space are presented in Fig. 2. The Euclidean distances on the map represent the similarity of the chemical fingerprints of the samples to each other. According to Fig. 3, it was observed that the sampling points were not randomly distributed, but rather formed four main clusters according to certain characteristics:

Group 1 (Sampling points 1, 4, and 6): Located very close to each other, these sampling points are situated around residential areas, parking lots, and shopping malls, indicating that the element load in this group is related to “stop-and-go” traffic. Parking lots and shopping mall entrances and exits are areas where metals such as Zn and Cu, which are particularly associated with tire and brake pad wear in the literature, accumulate heavily. This group represents pollution directly originating from vehicle traffic and urban activity.

Group 2 (Sampling points 2 and 3): Clearly separated from the other groups and forming a consistent cluster within itself, these sampling points represent a region dominated by natural vegetation, such as a dam basin, the Istanbul Metropolitan Municipality City Forest, and forested areas. Although located on the main artery, the dominance of the environmental matrix (soil/forest) has resulted in lithogenic (natural) elements (Al, Fe, Si) being more prominent at these sampling points, separating them from dense urban areas. This separation, as noted in the literature, is characteristic of areas where natural geological structure is dominant.

Group 3 (Sampling point 25): As the most striking finding of the analysis, sampling point 25, located in an isolated position from all other clusters, explains its "outlier" status due to its proximity to the quarry area and its location on the busy airport road traffic route. The intense dust (resuspension) from the quarries and the particulate matter load created by excavation trucks, combined with the heavy traffic emissions from the airport road, have created a specific and very high pollution profile at this point.

Group 4 (General Distribution—Urban Background): The large and central cluster formed by the remaining samples; It encompasses areas where residential and commercial areas are intertwined. This group represents the overall “urban background” pollution of the study area. A homogeneous mixture of traffic, heating, and commercial activities has made these sampling points chemically similar and grouped them into a common profile that is less specialized (not extreme values like groups 1 or 3).

### Pollution profiles

The results of the Two-Way Hierarchical Clustering Analysis (HCA), applied to determine the pollution profiles and spatial similarities of the sampling points based on their elemental content, show that the sampling points do not exhibit a random distribution, but rather clustered into three main groups according to distinct geochemical characteristics. When the resulting dendrogram structure and heatmap are evaluated together, these clusters are defined as “Clean/Background (C1)”, “Dirty (C2)” and “Transition (C3)” groups according to their pollution loads. Cluster 1 (C1) is characterized by the dominance of blue tones representing low concentrations in the heatmap and represents the natural background levels in the study area and the sampling points where anthropogenic influence is minimal (Si, Al, Fe, Mg, Ti). Cluster 2 (C2), determined as a result of the HCA analysis, brings together the sampling points with the highest concentrations of anthropogenic elements (Zn, Pb, Cu, Cd, Hg) (Jiang et al., 2020; Ulutaş, 2023b). However, as seen in the Spearman correlation analysis, the elements in this group are further subdivided. Cluster C2 encompasses ‘hotspots’ where both traffic-related (Zn–Pb–Cu) and combustion/industrial (Cd–Hg) pollution are concentrated. This indicates that C2 Sampling points are under the influence of multiple pollution sources rather than a single source. Cluster 3 (C3), on the other hand, is located between C1 and C2 in terms of element concentrations (Ba, V, Na, Mg, Mn, Mo, and Sb), showing that the pollution is distributed not with sharp boundaries, but within a spatial continuity and transitional structure.

**Fig. 4:**
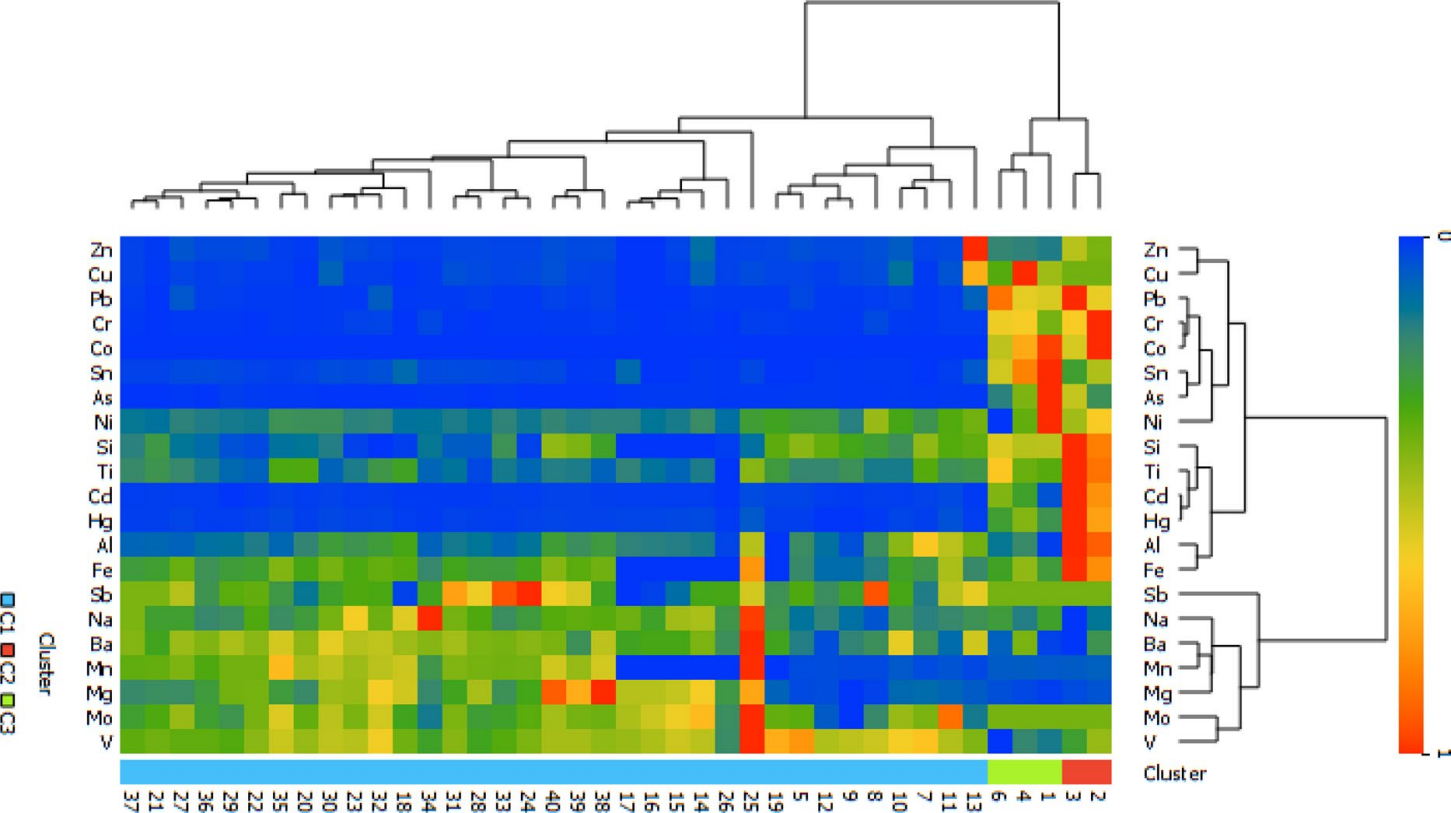
Hierarchical cluster analysis dendrogram and heatmap of elements and sampling sites. The figure presents the hierarchical cluster analysis results based on Ward’s linkage method and squared Euclidean distance. The heatmap shows standardized element concentrations across sampling sites. Dendrograms illustrate clustering patterns of elements and sampling sites. Color intensity represents relative concentration levels

HCA based on standardized element concentrations is presented in Fig. 4**.** The dendrogram classified the sampling sites into three clusters labeled C1, C2, and C3. Cluster C2 showed elevated standardized values for Co, Ni, Pb, Cr, As, Cd, and Hg across multiple sampling sites. Cluster C1 showed consistently lower standardized values for most elements. Cluster C3 exhibited intermediate standardized values between C1 and C2. Element-based clustering grouped Co, Ni, Pb, Cr, As, Cd, and Hg within the same dendrogram branch at low linkage distances, whereas Si, Al, Fe, Mg, Na, Mn, and Ba were separated into distinct branches. The separation of elements into two main clusters in the dendrogram proves that pollution sources are clearly differentiated. While the first main group (Si, Al, Fe, Mg) represents the natural geological structure (lithogenic origin), the second main group (Pb, Zn, Cd, Hg, Co) represents the pollution load where anthropogenic activities are dominant. Furthermore, the sub-branching within the contaminated group reflects the distinct pollution signatures of traffic and quarry activities (Ferreira-Baptista & De Miguel, 2005).

**Fig. 5:**
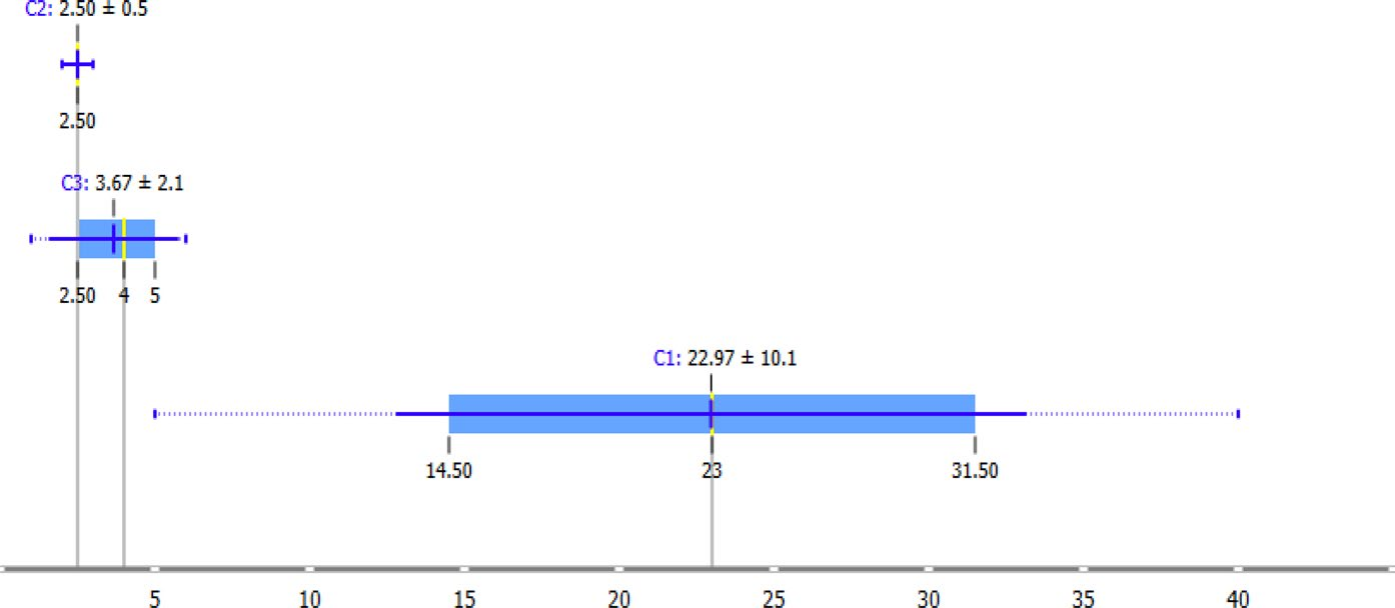
Box plots comparing the element concentration distributions of the identified pollution clusters (Clean/Background (C1), Dirty (C2) and Transition (C3))

The box plot distributions of the MDS dimension scores are shown in Fig. 5. The configuration plot resolved three clusters consistent with the hierarchical cluster analysis. Cluster C2 showed the highest mean score along the primary dimension (22.97 ± 10.1) and the widest dispersion range, spanning approximately 35 units. Cluster C1 showed the lowest mean score (2.50 ± 0.5) with a narrow dispersion range. Cluster C3 displayed an intermediate mean score (3.67 ± 2.1) and moderate dispersion. As illustrated in Fig. 5, the wide dispersion of C2 reflects pronounced heterogeneity among highly contaminated sites and indicates localized emission hotspots within the study area. Consistency between Figs. 3 and 5 demonstrates the robustness of the multivariate interpretation.

The differentiation of element concentrations among the groups identified by clustering analysis was examined using box plots. The plots visually confirm that group C2 has significantly higher median and upper quartile values, particularly for toxic metals (Pb, Cd, Hg, Zn), compared to other clusters. The wide range of variation and upper end values observed in group C2 demonstrate that metal accumulation at these sampling points is not homogeneous, but rather that point sources of pollution are dominant. This clear divergence on the box plot supports the idea that the classification performed with HCA is not merely the result of a statistical algorithm, but successfully reflects the actual pollution profiles in the field.

The statistical significance of the concentration differences between the identified clusters was analyzed using the non-parametric Kruskal–Wallis H test due to the non-normal distribution of the dataset (Table 4). The test results revealed statistically significant differences between clusters for the vast majority of the elements examined (*p* < 0.05). Specifically, the test statistics were calculated to be quite high (χ^2^ > 12.8) for Hg, Cd, Pb, Cr, Sn, Co, and As, and significantly higher (χ^2^ > 11.4) for traffic-derived Zn and Cu. The statistical significance level of the intergroup difference is high for all these anthropogenic elements (*p* < 0.01). The high effect size values (ε^2^, 0.33) calculated for these elements demonstrate that cluster membership is a decisive factor in explaining concentration variability. In contrast, no statistically significant difference was found between the groups for the elements Mn, Sb, and Mo, which are considered to be of lithogenic origin (Guo et al., 2024; Laznicka, 1992; Song et al., 2019) (*p* > 0.05). This indicates that the distribution of these elements follows a homogeneous course under the control of the natural geological structure, independent of pollution profiles (Duzgoren-Aydin et al., 2006). Consequently, the Kruskal–Wallis test confirmed the statistical reliability of the “Dirty Group (C2)” distinction determined by HCA and confirmed that metal pollution in the region originates from multiple anthropogenic sources.

**Table 4:** Kruskal–Wallis H test and effect size (ε^2^) results showing the statistical significance of the differences in element concentrations between clusters

Kruskal–Wallis				
Elements	χ^2^	df	*p*	ε^2^
Si	12.86	2	0.002	0.32974
Na	5.418	2	0.067	0.13892
Al	6.282	2	0.043	0.16107
Fe	6.282	2	0.043	0.16107
Mg	9.106	2	0.011	0.23348
Ti	12.314	2	0.002	0.31575
Mn	0.608	2	0.738	0.01558
Zn	11.438	2	0.003	0.29329
Ba	6.785	2	0.034	0.17398
Cu	11.673	2	0.003	0.2993
Cr	12.86	2	0.002	0.32974
V	9.518	2	0.009	0.24406
Pb	12.829	2	0.002	0.32896
Ni	5.787	2	0.055	0.1484
Sb	0.848	2	0.654	0.02175
Co	12.811	2	0.002	0.32849
As	12.805	2	0.002	0.32833
Cd	12.865	2	0.002	0.32986
Hg	12.95	2	0.002	0.33204
Mo	0.262	2	0.877	0.00672
Sn	12.861	2	0.002	0.32977

*p < 0.05 is statistically significant

### Correlation analysis

**Fig. 6:**
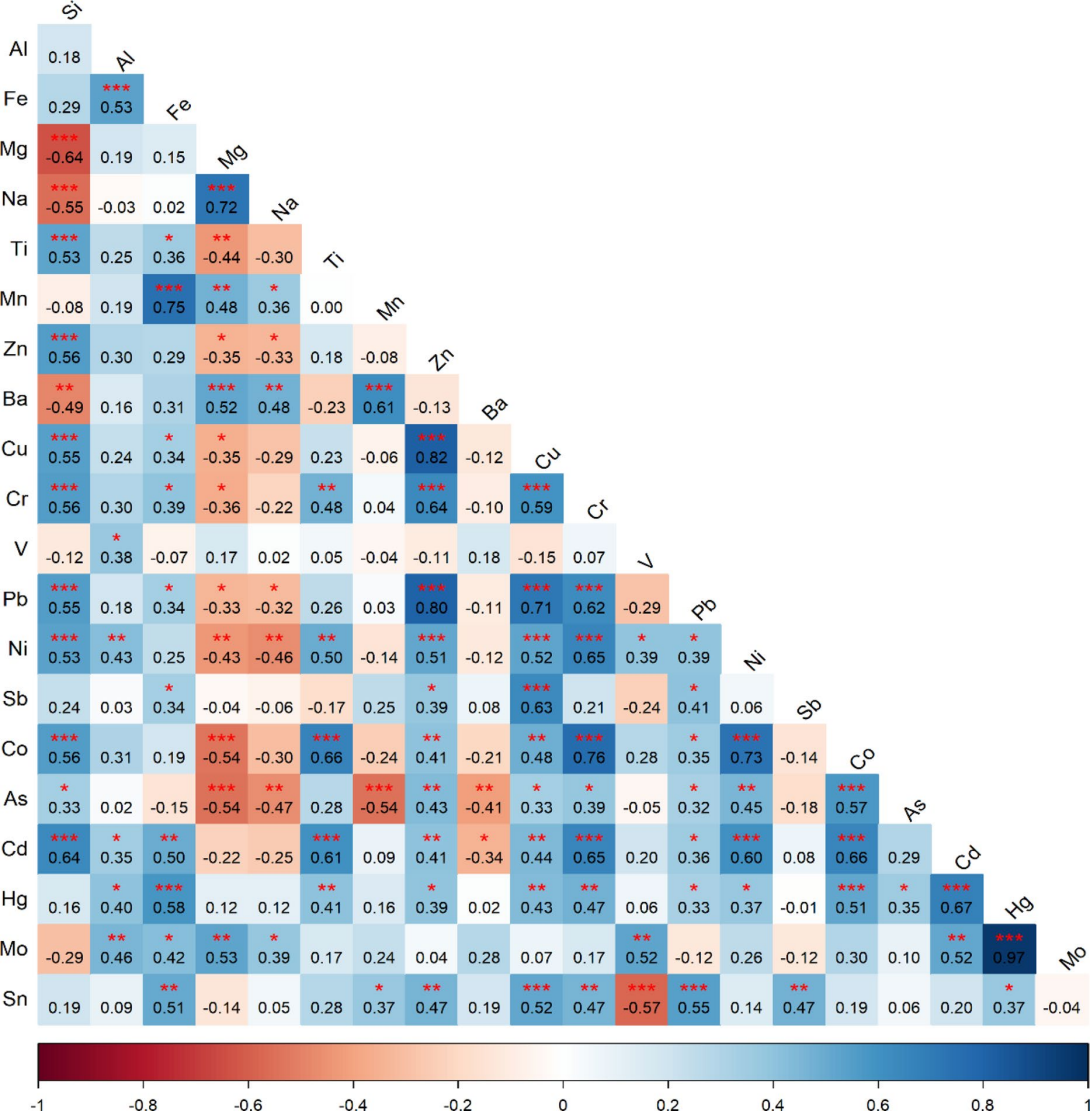
Results of Spearman correlation analysis between element concentrations measured in street dust samples. The matrix shows Spearman rank correlation coefficients between measured elements in street dust samples. Color intensity indicates the strength and direction of correlations. Positive correlations are shown in red and negative correlations in blue. Asterisks denote statistically significant correlations at *p* < 0.05

Spearman rank correlation coefficients between measured elements are presented in Fig. 6. Strong positive correlations were observed among Co, Ni, Pb, Cr, As, Cd, and Hg, with *r* values ranging from 0.73 to 0.98 (*p* < *0.05*). The highest correlation coefficient was detected between Cd and Hg (*r* = 0.98). Co showed strong positive correlations with Ni (*r* = 0.87), Pb (*r* = 0.93), and As (*r* = 0.86). Pb exhibited strong positive correlations with Cr (*r* = 0.91), Ni (*r* = 0.87), and Cd (*r* = 0.89). Cr was strongly correlated with Co (*r* = 0.72) and As (*r* = 0.63). As showed strong correlations with Cd (*r* = 0.73) and Hg (*r* = 0.81). Zn showed moderate positive correlations with Cu (*r* = 0.72) and Ba (*r* = 0.71). Cu was moderately correlated with Ba (*r* = 0.72) and Cr (*r* = 0.69). As shown in Fig. 6, the strong positive correlations among Co, Ni, Pb, Cr, As, Cd, and Hg indicate a shared source or closely linked input pathways, suggesting dominant anthropogenic control rather than independent geogenic contributions (Schiavo et al., 2023b; Skorbiłowicz et al., 2023). Negative correlations were observed between lithogenic elements and several potentially toxic elements. Si showed negative correlations with Mg (*r* = − 0.45) and Na (*r* = − 0.47). Mg was negatively correlated with Cr (*r* = − 0.38) and Pb (*r* = − 0.39). V showed negative correlations with Cr (*r* = − 0.41), Pb (*r* = − 0.54), and Co (*r* = − 0.53). The inverse relationships observed in Fig. 6 support source differentiation between lithogenic background elements and anthropogenic enriched metals (Ren et al., 2024).

The results of the Spearman rank correlation analysis show that the elements in the study area are divided into distinct groups according to their sources. In particular, the strong positive correlations observed between Zn–Cu (*r* = 0.82), Zn–Pb (*r* = 0.80), and Cu–Sb (*r* = 0.63) may indicate that these elements are released into the environment through a common mechanism related to intensive traffic activities (tire wear, brake pad debris, and exhaust emissions). In contrast, contrary to previous analyses, the element Hg (mercury) was found to exhibit very weak relationships with the traffic markers Pb (*r* = 0.33) and Zn (*r* = 0.16). The strongest correlation of Hg with Cd (*r* = 0.67) suggests that these two metals originate from a different anthropogenic source, such as fossil fuel (coal) combustion or specific industrial activities in the region, rather than traffic. The low correlation coefficients between lithogenically derived Al and toxic metals statistically support the idea that metal accumulation in the region is independent of natural geological processes.

### Pollution indices

**Fig. 7:**
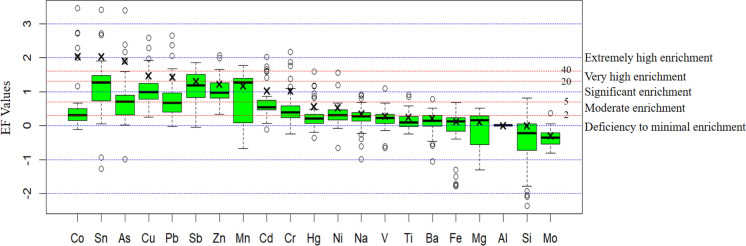
Enrichment factor (EF) values of heavy metals in street dust samples. Box plots present the distribution of enrichment factor values for individual heavy metals in street dust samples collected from the study area. Horizontal dashed lines indicate enrichment categories ranging from deficiency to minimal enrichment to extremely high enrichment. Boxes represent the interquartile range, horizontal lines denote median values, and crosses indicate mean values. The y-axis is logarithmic

Pollution indices were applied to quantify contamination intensity, enrichment characteristics, and ecological risk associated with the analyzed elements in street dust samples. Enrichment factor results further elucidate the origin and magnitude of elemental enrichment and are summarized in Fig. 7. Mo, Si, Mg, Al, Ba, Fe, Ti, V, and Na exhibited EF values below 2.00, indicating deficiency to minimal enrichment and a dominant lithogenic contribution (Wei & Yang, 2010). Moderate enrichment was observed for Ni and Hg, with EF values of 2.02 and 3.26, respectively. Cr, Mn, Cd, Zn, Sb, and Pb showed EF values ranging from 8.22 to 19.16, corresponding to significant to very high enrichment. Higher enrichment levels were recorded for Cu and As, with EF values of 20.99 and 35.03, respectively. The highest EF values were observed for Sn (57.04) and Co (63.45), indicating extremely high enrichment. Such elevated EF values demonstrate that Co and Sn are predominantly controlled by non-lithogenic sources and reflect strong anthropogenic inputs rather than natural geochemical variability (Nduka et al., 2024). Similar enrichment patterns have been documented in urban environments influenced by quarrying activities, where mechanical extraction, material handling, and dust resuspension processes enhance the accumulation of Co, Sn, and As bearing particles (Csavina et al., 2012).

**Fig. 8:**
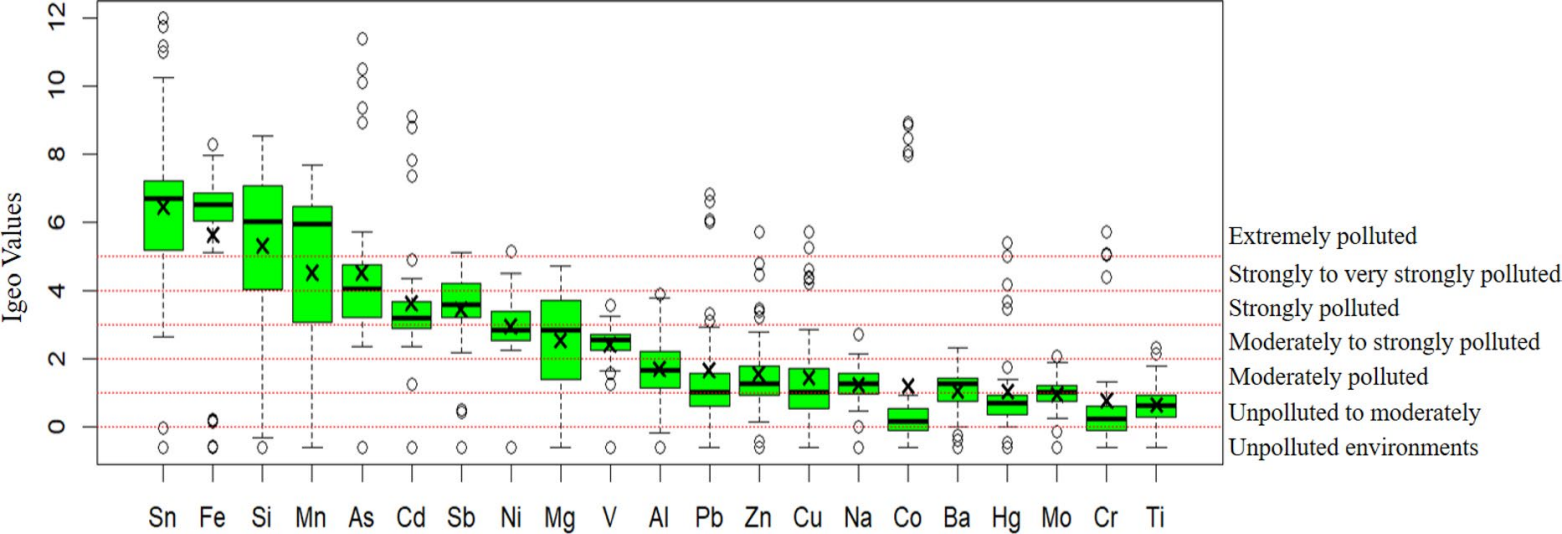
Geo-accumulation index (Igeo) values of heavy metals in street dust samples. Box plots show the distribution of Igeo values for individual heavy metals in street dust samples collected from the study area. Horizontal dashed lines represent contamination classes ranging from unpolluted to extremely polluted environments. Boxes indicate the interquartile range, horizontal lines represent median values, and crosses denote mean values

In the Igeo calculation, the minimum concentration value from the study was used as the background value (Chan et al., 2001) due to the possibility that it might not fully represent the natural structure (Verma et al., 2020). Igeo values are presented in Fig. 8 and indicate pronounced differences in contamination severity among elements. Al, Hg, Zn, Cr, V, and Cu exhibited I*geo* values ranging from 2.03 to 2.64, corresponding to moderately to strongly contaminated conditions. Mg, Ni, Pb, and Sb showed higher I*geo* values between 3.10 and 3.81, indicating strong contamination. The highest contamination levels were observed for Cd, Co, Mn, Si, Fe, As, and Sn, with I*geo* values ranging from 5.46 to 8.73, classifying these elements as extremely contaminated. Extremely high Igeo values for Co, Sn, and As indicate contamination levels far exceeding natural background concentrations, reflecting strong anthropogenic inputs in areas impacted by extractive activities (Howlader, 2025; Peng et al., 2016). Comparable I*geo* patterns have been reported in quarry-affected urban settings, where continuous deposition of mineral- and metal-bearing particles leads to persistent surface contamination (Rienda et al., 2025).

In this study, trace elements representing both natural and anthropogenic sources were analyzed in street dust samples collected across a wide area and in proximity to different emission sources. The Sultangazi district, where active quarries are located, was identified as the most affected area. Dust samples collected near these quarries showed significantly elevated concentrations of Si, Al, Fe, Cu, Cr, Pb, Co, As, Cd, and Sn compared to other sample points. This spatial gradient supports the role of quarry-derived particulate matter and indicates effective particulate transport from source areas. The fact that the samples were taken in June indicates the effect of a dry environment and atmospheric transport processes. Considering that the samples were collected from street dust, traffic emissions are an expected contributing factor; however, the pronounced enrichment of several elements indicates a substantial influence from quarry-derived dust. Elements such as Fe, Cu, and Sb, which are prominent in road dust, are typically associated with traffic-related sources (e.g., brake and tire wear), whereas crustal elements such as Si, Al, and Mg reflect natural and lithogenic inputs. Under dry conditions, fine particles generated by quarry operations can be easily mobilized, transported by wind, and deposited onto road surfaces. The simultaneous enrichment of both traffic-related and crustal elements therefore suggests a combined influence of vehicular emissions and quarry-derived particulate matter, contributing to the elevated concentrations observed in street dust. The effectiveness of industrial sources in element formation is particularly evident in the complete mixture of industrial and quarry-related elements. The resuspension of these dust particles over time and the role of wind transport create a truly matrix road dust. The accumulation of heavy metals (Pb, Zn, Cu, Cr) within this dust is an inevitable consequence.

### Human health risk assessment

Non-carcinogenic risk values for adults and children across inhalation, ingestion, and dermal contact pathways are presented in Table 5. In adults, ingestion-related HQ values ranged from 4.8E−04 for Hg to 1.7E+00 for Co. Dermal HQ values ranged from 5.1E−05 for Cu to 7.3E−02 for Co. Inhalation HQ values ranged from 4.9E−06 for Sb to 4.4E−02 for Co. HI values in adults exceeded 1 only for Co (1.8E+00). HI values for other elements ranged from 5.0E−04 for Hg to 1.8E + 00 for Co. The sum of HI values was 2.7E+00. In children, ingestion-related HQ values ranged from 5.1E−03 for Hg to 2.8E+00 for As. Dermal HQ values ranged from 3.1E−04 for Cu to 4.4E−01 for Co. Inhalation HQ values ranged from 2.6E−07 Hg to 9.0E−02 for Co. HI values in children exceeded 1 for As (2.8E+00), Cr (4.7E+00), and Co (1.9E+01). The highest HI value was recorded for Co. The sum of HI values was 2.8E+01.

**Table 5:** Non-carcinogenic and carcinogenic health risk assessment of potentially toxic elements in street dust

	Adults				Children				Adults	Children
PTEs	Inh.HQ	Ing.HQ	Derm.HQ	HI = ∑HQ	Inh.HQ	Ing.HQ	Derm.HQ	HI = ∑HQ	Inh.CR	Inh.CR
As	2.7E−03	2.6E−01	1.2E−03	2.7E−01	5.5E−03	2.8E+00	7.1E−03	2.8E+00	5.0E−08	3.1E−08
Co	4.4E−02	1.7E+00	7.3E−02	1.8E+00	9.0E−02	1.8E+01	4.4E−01	1.9E+01	6.9E−07	4.2E−07
Cr	6.1E−03	4.0E−01	6.7E−02	4.7E−01	1.2E−02	4.2E+00	4.0E−01	4.7E+00	5.8E−07	3.5E−07
Mn	4.2E−02	2.9E−02	2.1E−03	7.4E−02	8.6E−02	3.1E−01	1.2E−02	4.1E−01		
Ni	1.7E−03	4.1E−03	4.3E−04	6.2E−03	3.3E−03	4.4E−02	2.6E−03	4.9E−02	1.6E−09	9.6E−10
Pb		4.3E−02	1.8E−03	4.5E−02		4.6E−01	1.1E−02	4.7E−01	2.7E−10	1.7E−10
Sb	4.9E−06	7.2E−03	2.0E−04	7.4E−03	1.0E−05	7.7E−02	1.2E−03	7.8E−02		
Zn		1.5E−03	6.5E−05	1.6E−03		1.6E−02	3.9E−04	1.7E−02		
Cd	2.8E−05	5.5E−04	9.3E−05	6.8E−04	5.8E−05	5.9E−03	5.6E−04	6.5E−03	1.5E−10	8.9E−11
Cu		6.9E−03	5.1E−05	7.0E−03		7.4E−02	3.1E−04	7.4E−02		
Hg	1.3E−07	4.8E−04	2.9E−05	5.0E−04	2.6E−07	5.1E−03	1.7E−04	5.2E−03		
∑	9.7E−02	2.5E+00	1.5E−01	2.7E+00	2.0E−01	2.6E+01	8.8E−01	2.8E+01	1.3E−06	8.0E−07

PTEs potentially toxic elements,HQ hazard quotient, HI hazard index, Inh. inhalation exposure pathway, Ing. ingestion exposure pathway, Derm. dermal contact exposure pathway. HI represents the sum of HQ values across all exposure pathways for each element. CR carcinogenic risk. Carcinogenic risk was calculated for the inhalation exposure pathway

Carcinogenic risk values calculated for the inhalation pathway are presented in Table 5. In adults, CR values ranged from 1.5E−10 for Cd to 6.9E−07 for Co. CR values for As and Cr were 5.0E−08 and 5.8E−07, respectively. The sum of CR values was 1.3E−06 for adults. In children, CR values ranged from 8.9E−11 for Cd to 4.2E−07 for Co. CR values for As and Cr were 3.1E−08 and 3.5E−07, respectively. The sum of CR values was 8.0E−07 for children.

Human health risk assessment results indicate clear contrasts among exposure pathways and population groups. Non-carcinogenic risk estimates presented in Table 5 indicate that ingestion constitutes the dominant exposure pathway for adults and children, reflecting frequent contact with contaminated surface materials in densely populated urban environments. Similar dominance of ingestion-driven exposure has been reported for urban street dust in Ankara, where hand-to-mouth behavior and surface contact were identified as key contributors to non-carcinogenic risk, particularly for children (Gul et al., 2023; Isinkaralar et al., 2024). Higher HI values observed among children in this study are consistent with findings from Istanbul, where age-specific exposure characteristics contribute to increased vulnerability among children living in traffic- and activity-intensive districts (Öncü et al., 2025).

The substantially higher HI values observed for children reflect increased vulnerability associated with higher ingestion rates and lower body weight, as commonly reported in urban exposure assessments involving contaminated dust (Isinkaralar et al., 2024). Similar age-dependent differences in non-carcinogenic risk have been reported in quarry-influenced environments, where dust-mediated exposure disproportionately affects children (Chamdimba et al., 2023). The dominance of the ingestion pathway reported in Table 5 suggests that direct contact with contaminated surface materials represents the principal exposure route in densely populated urban environments.

All calculated CR values remained within the acceptable risk range, indicating the absence of immediate carcinogenic concern based on inhalation exposure. The cumulative CR values underscore the relevance of long-term exposure assessment in urban districts characterized by mixed anthropogenic sources, where incremental contributions from multiple elements may become environmentally significant over extended periods (Howlader, 2025). Similar carcinogenic risk patterns have been reported in rapidly urbanizing areas influenced by combined traffic and industrial emissions, supporting the importance of continued monitoring of metal-bearing street dust (Ren et al., 2024). The systematic evaluation and implementation of appropriate dust control techniques in quartering operations are essential for safeguarding occupational and public health. In addition, the effective enforcement of dust control measures plays a crucial role in mitigating the environmental impacts of quarrying activities, minimizing occupational exposure, and protecting public health (Saka & Hashim, 2024).

In this study, street dust exhibited high health hazard index values for Co, Cr, and As. In addition to other sources, the continuous and substantial generation of quarry dust associated with natural resource extraction in the study area appears to increase the local dust load, suggesting that these heavy metals may significantly contribute to human exposure. Prolonged exposure to elevated levels of street dust may also lead to respiratory diseases. For example, increased silicon concentrations in inhaled dust are associated with a higher risk of silicosis in humans. Furthermore, heavy metals are known to pose various health risks upon ingestion, including neurological impairment, respiratory disorders, hematological alterations, kidney dysfunction, and skin diseases. Therefore, additional measures should be implemented to reduce exposure to street dust.

## Conclusion

Providing the necessary gravel for urban redevelopment while controlling particulate matter from granite quarries through sustainable mining practices is crucial for environmental protection. This study investigates the elemental characterization of street dust and the associated human health risks in Sultangazi and the neighboring districts, urban areas of Istanbul with high population density and intensive traffic and quarry activity. The assessment included element concentrations measured by ICP-OES, pollution levels analyzed by enrichment factor (EF) and geoaccumulation index (Igeo), spatial analysis by GIS, and human health risk assessment for both adults and children using a methodology developed by the USEPA. Results indicate substantial metal accumulation in street dust. Mean concentrations reached 432.64 mg kg^−1^ for Co, 298.17 mg kg^−1^ for Cr, 384.08 mg kg^−1^ for Zn, 128.38 mg kg^−1^ for Pb, and 66.27 mg kg^−1^ for As. EF values exceeded 40 for Co and Sn, and I*geo* classified Co, As, Cd, and Sn as extremely contaminated. GIS analysis identified pronounced spatial heterogeneity and discrete contamination hotspots aligned with high-traffic corridors and quarry proximity. Health risk metrics indicate clear age-related contrasts. Among the routes of exposure, ingestion dominated in non-carcinogenic health risks. HI values exceeded unity for children and reached 2.8 for As, 4.7 for Cr, and 19.0 for Co, indicating unacceptable non-carcinogenic risk at several sampling locations. Carcinogenic risk values remained within accepted limits for both age groups, and inhalation exposure remains relevant under continuous contact conditions. Based on these technical parameters, the implementation of measures to control dust from quarries should be considered not merely a luxury endeavor, but a vital matter for protecting the health of workers in this sector, promoting the well-being of communities living near quarries, and conserving the environment. The quantitative outcomes provide a basis for identifying priority zones requiring intervention, particularly in areas where traffic activity and quarry operations overlap. These results can inform site-specific dust control measures, exposure reduction strategies for vulnerable populations, and urban environmental planning decisions in quarry-influenced districts. Future studies may expand spatial coverage, incorporate seasonal variability, and refine background reference selection to improve source attribution and exposure characterization. Remote sensing methods, on-site health screenings, and close examination of health data of people living in the area are crucial for investigating specific health issues caused by quarry dust exposure and protecting environmental and public health. Particulate emissions from quarrying operations pose a significant environmental challenge, impacting ecosystems, air quality, and soil biota, and highlight the need for effective environmental and public health management strategies.

## Supplementary Information

Below is the link to the electronic supplementary material.

Supplementary file1 (DOCX 204 KB)

## Data Availability

Data will be made available on reasonable request.
