# Supplementary material for: Health risk assessment and geochemical characterisation of trace elements in street dust: a case study from a quarry-influenced urban area of Istanbul
Source: Environ Geochem Health. 2026 May 23;48(8):370. doi: 10.1007/s10653-026-03264-y (PMC13198513; doi:10.1007/s10653-026-03264-y)
Supplement: Supplementary file 1 — Supplementary file1 (DOCX 204 KB) [file 10653_2026_3264_MOESM1_ESM.docx]

**Health Risk Assessment and Geochemical Characterisation of Trace Elements in Street Dust: A Case Study from a Quarry-Influenced Urban Area of Istanbul**

Kadir Ulutaş^1^*, Elif Yavuz^1^, Hedanur Yıldız^1^, Rabia Emlek^1^, Şilan Tekin^1^, Didar Üçüncüoğlu^2^, Emre Yücer^3^, Enes Özkök^4^, Orhan Sevimoğlu^5^, Abdulkadir Keskin^6^, Seda Uyar^1^

^1^ İstanbul Medeniyet University, Faculty of Health Sciences, Department of Health Management, Istanbul, Türkiye

^2^ Çankırı Karatekin University, Faculty of Engineering, Food Engineering Department, Çankırı, Türkiye

^3^ University of Karabük, TOBB Vocational School of Technical Sciences, Karabük, Türkiye

^4^ University of Karabük, Faculty of Engineering, Department of Environmental Engineering, Karabük, Türkiye

^5^ Gebze Technical University. Faculty of Engineering, Department of Environmental Engineering, Kocaeli. Türkiye

^6^ Istanbul Medeniyet University, Faculty of Engineering and Natural Sciences, Department of Statistics, Istanbul, Türkiye

*Corresponding author: Kadir Ulutaş (E-mail: [kadir.ulutas@medeniyet.edu.tr](mailto:kadir.ulutas@medeniyet.edu.tr))

**SUPPLEMENTARY INFORMATION FOR THE RESEARCH PAPER TITLED:**

**Table S1.** Data regarding quality control and quality assurance for some elements.

|  | Analysis Data  (mg kg^-1^) | Certified Value  (mg kg^-1^) | Uncertainly | Recovery, % | SD | LOD  ppb | LOQ  ppb |
| --- | --- | --- | --- | --- | --- | --- | --- |
| Al | 38650 | 37500 | 0.22 | 103.07 | 15.36 | 46.08 | 153.6 |
| Ba | 465 | 460 | 0.04 | 101.09 | 2.256 | 6.768 | 22.56 |
| Cd | 2.754 | 2.5 | 0.4 | 110.16 | 0.279 | 0.837 | 2.79 |
| Co | 28.563 | 29.8 | 1.6 | 95.85 | 0.235 | 0.705 | 2.35 |
| Cr | 432.596 | 440 | 18 | 98.31 | 0.321 | 0.963 | 3.21 |
| Fe | 31560 | 32900 | 0.2 | 95.94 | 20.35 | 61.05 | 203.5 |
| Mn | 1562 | 1280 | 0.04 | 122.03 | 5.36 | 16.08 | 53.6 |
| Mo | 39.845 | 40 | 0.6 | 99.61 | 0.124 | 0.372 | 1.24 |
| Ni | 178.542 | 171 | 3 | 104.41 | 0.214 | 0.642 | 2.14 |
| Pb | 888.653 | 866 | 16 | 102.61 | 0.256 | 0.768 | 2.56 |
| Sb | 30.25 | 28.2 | 2.3 | 107.25 | 0.321 | 0.963 | 3.21 |
| Ti | 2.657 | 2.58 | 0.13 | 102.98 | 3.256 | 9.768 | 32.56 |
| V | 72.569 | 74.9 | 1.9 | 96.89 | 0.412 | 1.236 | 4.12 |
| Zn | 1526 | 1660 | 0.1 | 91.93 | 0.125 | 0.375 | 1.25 |

**Table S2.** Description and values of parameters for average daily intake (USEPA 2014; USEPA 2002; Bartholomew et al. 2020)

| Parameters | Description | Unit | Adults | Children |
| --- | --- | --- | --- | --- |
| C | Concentration of PTEs | mg·kg^-1^ |  |  |
| EF | Exposure frequency | d·year^-1^ | 350 | 350 |
| ED | Exposure duration | year | 20 | 6 |
| IngR | Ingestion rate | mg·day^-1^ | 100 | 200 |
| AT (Carcinogenic) | Average time | day | 365×70 | 365×70 |
| AT (Non-carcinogenic) | Average time | day | 365×20 | 365×6 |
| BW | Average body weight | kg | 80 | 15 |
| InhR | Inhalation rate | m^3^·day^-1^ | 20 | 7.63 |
| PEF | Particle emission factor | m^3^·kg^-1^ | 1.36×10^9^ | 1.36×10^9^ |
| AF | Adherence factor | mg·cm^-2^ | 0.07 | 0.2 |
| SA | Skin area | cm^2^ | 6032 | 2373 |
| ABS | Dermal absorption | dimensionless | 0.001 | 0.001 |

**Table S3.** The Reference Dose [RfD, (mg/kg·day)] and Cancer Slope Factor [SF, (kg.day/mg)] of PTEs via three exposure pathways (Inhalation, Ingestion, and Dermal Contact) (Integrated Risk Information System, 2025; USEPA, 2002; Konstantinova et al., 2022).

| PTEs | Inhalation RfD | Ingestion RfD | Dermal RfD | Inhalation CSF |
| --- | --- | --- | --- | --- |
| As | 4.3E-06 | 3.0E-04 | 2.9E-04 | 1.5E+01 |
| Co | 1.7E-06 | 3.0E-04 | 3.0E-05 | 3.2E+01 |
| Cr | 8.6E-06 | 9.0E-04 | 2.3E-05 | 3.9E+01 |
| Mn | 1.4E-05 | 1.4E-01 | 8.4E-03 |  |
| Ni | 4.0E-06 | 1.1E-02 | 4.4E-04 | 8.4E-01 |
| Pb |  | 3.6E-03 | 3.6E-04 | 4.2E-02 |
| Sb | 8.6E-05 | 4.0E-04 | 6.0E-05 |  |
| Zn |  | 3.0E-01 | 3.0E-02 |  |
| Cd | 2.9E-06 | 1.0E-03 | 2.5E-05 | 6.3E+00 |
| Cu |  | 4.0E-02 | 2.3E-02 |  |
| Hg | 8.6E-05 | 1.6E-04 | 1.1E-05 |  |

**References**

Bartholomew, C. J., Li, N., Li, Y., Dai, W., Nibagwire, D., & Guo, T. (2020). Characteristics and health risk assessment of heavy metals in street dust for children in Jinhua, China. Environmental Science and Pollution Research, 27(5), 5042–5055.

Konstantinova, E., Minkina, T., Konstantinov, A., Sushkova, S., Antonenko, E., Kurasova, A., & Loiko, S. (2022). Pollution status and human health risk assessment of potentially toxic elements and polycyclic aromatic hydrocarbons in urban street dust of Tyumen city, Russia. Environmental Geochemistry and Health, 44(2), 409-432.

USEPA. (2002). Supplemental Guidance for developing soil screening levels for superfund sites. Office of Solid Waste and Emergency Response. OSWER 9355.4-24 Washington DC.

USEPA. (2014). Human health evaluation manual, supplemental guidance: Update to standard default exposure factors. (OSWER 9200.1-120). Washington, DC: National Center for Environmental assessment.

USEPA. (2025). United States Environmental Protection Agency. Integrated Risk Information System. <https://iris.epa.gov/AtoZ/?list_type=alpha>. Accessed 14 February 2026

a)


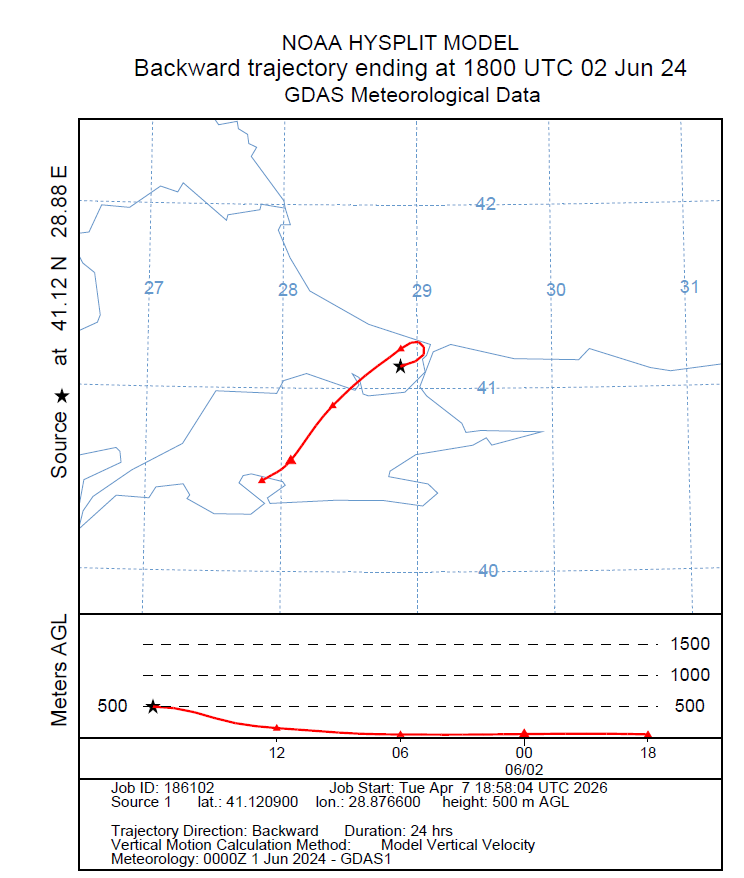


b)


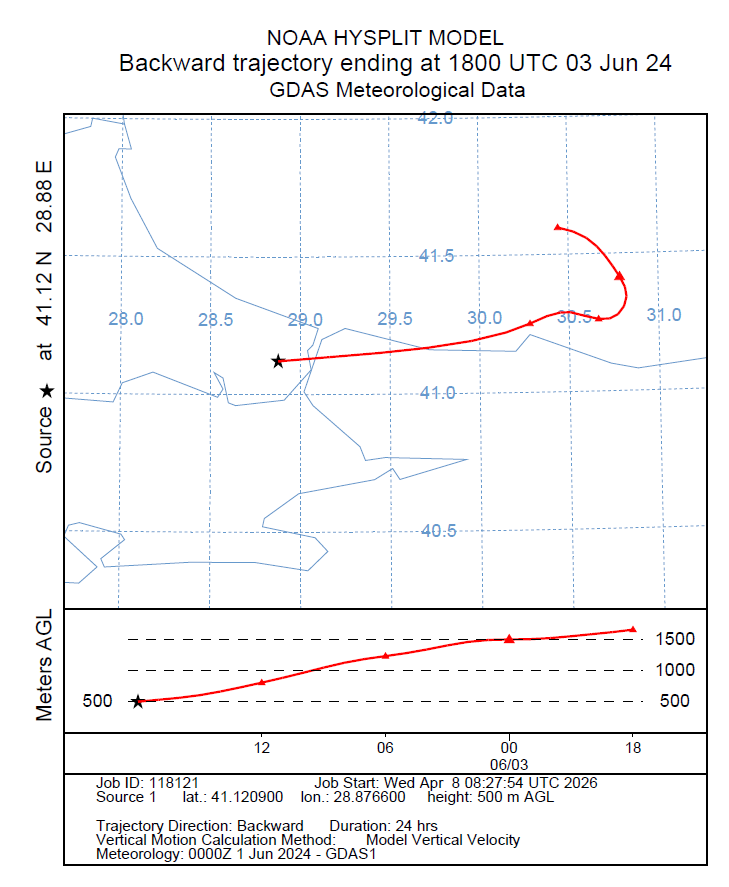


Fig. S1: HYSPLIT back trajectory demonstrate the wind direction on this days of June 2, 2024 (a) and June 3, 2024 (b).
